# Transitions in health care settings for frequent and infrequent users of emergency departments: a population-based retrospective cohort study

**DOI:** 10.1186/s12913-023-10260-w

**Published:** 2023-11-14

**Authors:** Rhonda J. Rosychuk, Anqi A. Chen, Maria B. Ospina, Andrew D. McRae, X. Joan Hu, Patrick McLane

**Affiliations:** 1 Department of Pediatrics, University of Alberta, Edmonton, AB T6G 1C9 Canada; 2 Department of Statistics and Actuarial Science, Simon Fraser University, Burnaby, BC V5A 1S6 Canada; 3 Department of Public Health Sciences, Queen’s University, Kingston, ON K7L 3N6 Canada; 4 Department of Emergency Medicine, University of Calgary, Calgary, AB T2N 2T9 Canada; 5 Department of Community Health Sciences, University of Calgary, Calgary, AB T2N 4Z6 Canada; 6 Emergency Strategic Clinical Network, Alberta Health Services, Edmonton, AB T5J 3E4 Canada; 7 Department of Emergency Medicine, University of Alberta, Edmonton, AB T6G 2R7 Canada

**Keywords:** Emergency department, Frequent health service users, Multistate models

## Abstract

**Background:**

Efforts to reduce emergency department (ED) volumes often target frequent users. We examined transitions in care across ED, hospital, and community settings, and in-hospital death, for high system users (HSUs) compared to controls.

**Methods:**

Population-based databases provided ED visits and hospitalizations in Alberta and Ontario, Canada. The retrospective cohort included the top 10% of all the ED users during 2015/2016 (termed HSUs) and a random sample of controls (4 per each HSU) from the bottom 90% per province. Rates of transitions among ED, hospitalization, community settings, and in-hospital mortality were adjusted for sociodemographic and ED variables in a multistate statistical model.

**Results:**

There were 2,684,924 patients and 579,230 (21.6%) were HSUs. Patient characteristics associated with shorter community to ED transition times for HSUs included Alberta residence (ratio of hazard ratio [RHR] = 1.11, 95% confidence interval [CI] 1.11,1.12), living in areas in the lower income quintile (RHR = 1.06, 95%CI 1.06,1.06), and Ontario residents without a primary health care provider (RHR = 1.13, 95%CI 1.13,1.14). Once at the ED, characteristics associated with shorter ED to hospital transition times for HSUs included higher acuity (e.g., RHR = 1.70, 95% CI 1.61, 1.81 for emergent), and for many diagnoses including chest pain (RHR = 1.71, 95%CI 1.65,1.76) and gastrointestinal (RHR = 1.66, 95%CI 1.62,1.71). Once admitted to hospital, HSUs did not necessarily have longer stays except for conditions such as chest pain (RHR = 0.90, 95% CI 0.86, 0.95). HSUs had shorter times to death in the ED if they presented for cancer (RHR = 2.51), congestive heart failure (RHR = 1.93), myocardial infarction (RHR = 1.53), and stroke (RHR = 1.84), and shorter times to death in-hospital if they presented with cancer (RHR = 1.29).

**Conclusions:**

Differences between HSUs and controls in predictors of transitions among care settings were identified. Co-morbidities and limitations in access to primary care are associated with more rapid transitions from community to ED and hospital among HSUs. Interventions targeting these challenges may better serve patients across health systems..

**Trial registration:**

Not applicable.

**Supplementary Information:**

The online version contains supplementary material available at 10.1186/s12913-023-10260-w.

## Background

Many urban emergency departments (EDs) face crowding issues with long wait times for patients [1]. High system users (HSUs) of EDs have been identified as a potential target of interventions to address crowding due to their disproportionately high numbers of ED presentations for a relatively small proportion of all ED users [2, 3]. Lacalle and Rabin cite literature showing that “Frequent users comprise 4.5% to 8% of all ED patients but account for 21% to 28% of all visits.” In the Canadian province of British Columbia, Moe and colleagues found that HSUs made up 15.8% of all ED patients but 40.3% of all ED visits. They showed that HSU’s use of ED was growing over time, and growing faster than non-HSUs ED use [4]. In pursuit of healthcare resource optimization, some look to the possibility of better serving HSUs [5]. This may occur by resolving reasons for frequent ED use in ED, or by linking frequent users to appropriate social and healthcare supports provided in other settings. Reduced ED visits might lead to reduced pressures on emergency providers and thus improvements in quality of care. Potential cost savings are also large, especially as frequent users of ED are also higher users of other costly health services including inpatient and ambulance [6].

Most studies on high ED use summarize characteristics of patients or ED presentations [3, 7–9]. Many authors estimate rates or expected counts using Poisson or negative binomial modeling [10–13]. Published studies tend to exclude other health services use like hospitalizations. Those analyses ignore the timing of events and thus cannot demonstrate the interconnections of ED presentations with other health services use. Multistate models deconstruct longitudinal data into distinct states that capture changes in health status over time [14, 15]. For example, states could be healthy, diseased, or dead and transitions are made between the healthy and diseased states as well as from the healthy state to dead and the diseased state to dead. Multistate models have the potential to identify factors associated with changes in health states and may lead to targeted interventions to increase or decrease the transition times.

**Fig. 1:**
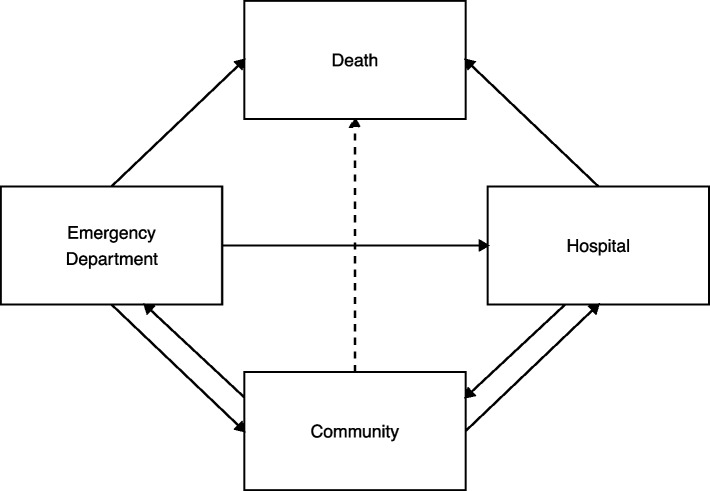
A four state model for health care states (solid arrows represent transitions observed and estimated)

The top 10% of patients with respect to the number of ED presentations during the fiscal year [16] form the HSU groups in two provinces for this study. We compare trends in ED presentations and hospitalizations for HSUs of EDs and controls using a multistate model. We deconstruct an individual’s time into mutually exclusive states, i.e. settings of in the ED, admitted to hospital, death in-ED or in-hospital, and in the community as illustrated in Fig. 1. The effects of covariates on transitions among states are examined via the multistate model. In particular, we determine if the covariate effects differ for HSUs and controls. A better understanding of the timing of ED presentations and hospitalizations may signal changes in health and help identify opportunities for interventions.

## Methods

### Study design

This retrospective study used data from population-based administrative health databases from the Canadian provinces of Alberta (population > 4 million [17]) and Ontario (population nearly14 million [17]) during April 1, 2015, to March 31, 2016. The study data are part of a larger data extract with similar methods and the characteristics of patients and ED presentations have already been described [6, 18]. The University of Alberta Health Research Ethics Board approved this study.

### Study setting and population

A dynamic cohort of the most frequent adult ED users [16] was created in collaboration between the Canadian Institutes of Health Research (CIHR) and the Canadian Institute for Health Information (CIHI). Alberta and Ontario are the only provinces that report on all ED presentations to the National Ambulatory Care Reporting System (NACRS) [16, 19] and CIHI holds and used this database. For each province, the top 10% of patients with respect to the number of ED presentations during the fiscal year [16] form the HSU groups. Control groups were also created for each province by selecting a random sample of patients not in the HSU groups during the fiscal year using a sampling ratio of 4:1 [16]. These design choices were made by CIHR and CIHI.

### Study protocol

The NACRS database provides demographic characteristics and ED presentation characteristics including dates and times, triage level, diagnoses, and disposition status. For Ontario, the data include access to primary health care provider. Triage level represents the urgency of ED care required and is based on the Canadian Emergency Department Triage and Acuity Scale (CTAS) [20, 21]. The triage codes are as follows: 1 = resuscitation, 2 = emergency, 3 = urgent, 4 = semi-urgent, and 5 = non-urgent, and grouped for this study as CTAS 1/2 vs. all others. Up to 10 diagnoses are provided as International Classification of Diseases (ICD-10-CA) [22] codes. The main diagnosis code was categorized using a combination of the Quan adaptation [23] to the Deyo/Charlson comorbidity coding scheme icd [24] and Guttmann et al.’s classification [25]. Any overlapping ICD codes were kept only in the Quan scheme to ensure mutually exclusive categories (Supplementary Table 1, Additional File 1). We grouped the 15 disposition codes as discharges, admissions/transfers, deaths, left without being seen (LWBS), and left against medical advice (LAMA).

The Discharge Abstract Database (DAD) [26] is also held by CIHI and provided hospitalization data (e.g., date/time of admission and discharge, ICD-10-CA [22] diagnosis codes). Statistics Canada 2011 census data provided neighborhood income quintiles and the population centre type (which depends on technical definitions of population size, census metropolitan area [CMA] and census agglomeration [CA] designations) [27]. We classified the area of residence into large urban areas (grouped from the categories of core, secondary core, and population centres outside CMA/CAs), fringe (all small urban areas within a CMA or CA that are not contiguous with the core of the CMA or CA), and rural [28].

#### Key outcome measures

We assume an individual is in one of four mutually exclusive states at any time during the study period (Fig. 1). The ED state defines the time in which patients are in the ED. The start of the ED state is the date and time (hour:minute) the patient arrives at the ED and the end of the ED state is that date and time a patient leaves the ED. The hospital state defines the time that patients are admitted to hospital. The community state defines the time that patients are neither in the ED nor admitted to hospital and have not died in those settings. The death state is limited to death in the ED or in the hospital (e.g., in-hospital death) because provincial vital statistics data are not part of CIHI holdings and linkage is not allowed. The rates of transition between states are the outcomes of interest.

### Data analysis

Summary statistics (e.g., sample means, sample standard deviations [SDs]) describe data. We excluded ED presentations and hospitalizations that were not entirely within the study period. There were some variables that had missing or not applicable categories (e.g., population type) and these were combined into a category referred to as missing. Statistical analyses were conducted in R [29] using the packages survival [30] and mstate [31, 32].

The mstate package was used to create the multistate model data structure assuming each transition time follows a Cox proportional hazards regression model, which formulates the distribution of a transition time as a function of state-specific covariates. The time associated with a transition to other states that does not occur is viewed as right-censored. All transitions included province, sex, age, lowest income quintile indicator, population centre type, and access to primary health care provider (Ontario only) as covariates. For transition from ED to hospital, triage level and diagnosis in the ED were additional covariates. For transitions from the ED to community state, ED diagnosis was included whereas for the hospital to community transition, hospital diagnosis was included. The time from last entry to community state to the end of study period was removed from analysis because death dates in the community were not known. Models included interactions with HSU status to determine if the effect of covariates on transitions was different for HSUs compared to controls. By nature of the HSU definition, the transition from community to ED is more likely but any differential effects of covariates nonetheless require quantification. Further, because data on access to primary health care provider is only available for Ontario patients, that covariate is only entered into the model as an interaction with an Ontario indicator variable. To adjust for correlated data (i.e., a patient may have multiple community to ED transitions), the nonparametric cluster bootstrap was employed with 100 samples with replacement [33]. Hazard ratios (HRs), ratios of hazard ratios (RHRs; the ratio of HSU HR and control HRs), and associated bootstrap percentile-based 95% confidence intervals (CIs) [34] are provided. Since interest focuses on differences between effects for HSUs and controls, we report RHRs. RHRs > 1 indicate a shorter time to transition for HSUs (shorter state occupancy) whereas RHRs < 1 indicate a longer time to transition for HSUs. Estimates of cumulative hazard functions are provided in Additional File 1. A *p*-value (p) less than 0.05 was considered to be statistically significant.

## Results

### Characteristics

There were 5,022,536 ED presentations and 724,369 hospitalizations available for analysis involving 2,684,924 patients. High system users were 579,230 (21.6%) of the patients and had 2,461,004 (49.0%) of the ED presentations and 388,578 (53.6%) of the hospitalizations (Table 1). About 80% of the hospital encounter data were from Ontario. The cohort had a large proportion of patients from large urban areas and from lower income areas. Over half of the transitions from ED to hospital and hospital to community were from HSUs but only 37.2% of community to hospital transitions were from HSUs. Most transitions to hospital went through the ED rather than directly from the community. About 38,000 patients died in the ED (29.3% HSUs) or during their hospital admission (46.2% HSUs). Characteristics by provinces and transitions also appear in Supplementary Tables 2–4, Additional File 1. There were important differences in the distribution of diagnoses among the various transitions (Supplementary Table 5, Additional File 1).

**Table 1:** Characteristics of patients and events at the different transitions

	Community to ED *n* = 5,022,536		ED to Community *n* = 4,446,600		ED to Hospital *n* = 571,556		Hospital to Community *n* = 690,159		Community to Hospital *n* = 152,813		ED to Death *n* = 4,380		Hospital to Death *n* = 34,210	
Distinct patients, n	2,683,716		2,509,757		410,411		505,973		146,599		4,380		34,210	
Group, n (%)														
Control	2,561,532	(51.0)	2,318,565	(52.1)	239,871	(42.0)	317,391	(46.0)	95,920	(62.8)	3,096	(70.7)	18,400	(53.8)
HSU	2,461,004	(49.0)	2,128,035	(47.9)	331,685	(58.0)	372,768	(54.0)	56,893	(37.2)	1,284	(29.3)	15,810	(46.2)
Province, n (%)														
Alberta	1,045,504	(20.8)	927,803	(20.9)	116,967	(20.5)	145,434	(21.1)	33,966	(22.2)	734	(16.8)	5,499	(16.1)
Ontario	3,977,032	(79.2)	3,518,797	(79.1)	454,589	(79.5)	544,725	(78.9)	118,847	(77.8)	3,646	(83.2)	28,711	(83.9)
Sex, n (%)														
Female	2,701,067	(53.8)	2,408,267	(54.2)	291,215	(51.0)	374,298	(54.2)	99,136	(64.9)	1,585	(36.2)	16,053	(46.9)
Male	2,321,469	(46.2)	2,038,333	(45.8)	280,341	(49.0)	315,861	(45.8)	53,677	(35.1)	2,795	(63.8)	18,157	(53.1)
Age (years)														
Mean (SD)	50.1	(20.5)	48.2	(19.9)	64.5	(19.8)	61.1	(20.5)	51.9	(19.9)	68.8	(17.4)	75.6	(14.0)
Median (Q1, Q3)	49.0	(32.0,66.0)	47.0	(31.0,63.0)	68.0	(52.0,81.0)	64.0	(46.0,78.0)	53.0	(33.0,69.0)	71.0	(58.0,83.0)	78.0	(67.0,86.0)
Neighborhood Income Quintile, n (%)														
Lowest	1,286,088	(25.6)	1,129,971	(25.4)	155,008	(27.1)	180,668	(26.2)	34,891	(22.8)	1,109	(25.3)	9,231	(27.0)
Medium–low	1,061,115	(21.1)	937,126	(21.1)	123,070	(21.5)	147,192	(21.3)	31,835	(20.8)	919	(21.0)	7,713	(22.5)
Middle	975,260	(19.4)	866,087	(19.5)	108,293	(18.9)	132,767	(19.2)	30,933	(20.2)	880	(20.1)	6,459	(18.9)
Medium–high	874,468	(17.4)	780,594	(17.6)	93,153	(16.3)	116,394	(16.9)	28,777	(18.8)	721	(16.5)	5,536	(16.2)
Highest	759,791	(15.1)	674,003	(15.2)	85,124	(14.9)	105,478	(15.3)	25,299	(16.6)	664	(15.2)	4,945	(14.5)
Missing / Not applicable	65,814	(1.3)	58,819	(1.3)	6,908	(1.2)	7,660	(1.1)	1,078	(0.7)	87	(2.0)	326	(1.0)
Population Type^a^, n (%)														
Large urban	3,663,357	(72.9)	3,220,533	(72.4)	439,596	(76.9)	526,037	(76.2)	112,623	(73.7)	3,228	(73.7)	26,182	(76.5)
Fringe	134,208	(2.7)	119,865	(2.7)	14,192	(2.5)	17,612	(2.6)	4,291	(2.8)	151	(3.4)	871	(2.5)
Rural area	830,521	(16.5)	750,840	(16.9)	78,968	(13.8)	99,188	(14.4)	25,362	(16.6)	713	(16.3)	5,142	(15.0)
Missing (not linked to geographic level or not applicable)	394,450	(7.9)	355,362	(8.0)	38,800	(6.8)	47,322	(6.9)	10,537	(6.9)	288	(6.6)	2,015	(5.9)
Primary Health Care (Ontario only), n (%)														
Family Physician	3,561,846	(89.6)	3,131,906	(89.0)	426,819	(93.9)	511,776	(94.0)	112,100	(94.3)	3,121	(85.6)	27,143	(94.5)
None	293,541	(7.4)	275,383	(7.8)	17,918	(3.9)	21,192	(3.9)	4,156	(3.5)	240	(6.6)	882	(3.1)
Other (ex. Family Health Team, Walk-in Clinic)	31,777	(0.8)	29,378	(0.8)	2,382	(0.5)	3,023	(0.6)	774	(0.7)	17	(0.5)	133	(0.5)
Unknown/Unavailable/ Missing (includes cases where patient is unconscious or arrives dead)	89,868	(2.3)	82,130	(2.3)	7,470	(1.6)	8,734	(1.6)	1,817	(1.5)	268	(7.4)	553	(1.9)
Triage Level, n (%)														
1- Resuscitation			12,091	(0.3)	27,260	(4.8)					3,444	(78.6)		
2-Emergent			703,884	(15.8)	249,311	(43.6)					614	(14.0)		
3-Urgent			1,935,178	(43.5)	257,514	(45.1)					199	(4.5)		
4-Semi-urgent			1,486,354	(33.4)	31,333	(5.5)					34	(0.8)		
5-Non-urgent			267,176	(6.0)	2,844	(0.5)					45	(1.0)		
Missing/unavailable			41,917	(0.9)	3,294	(0.6)					44	(1.0)		

*HSU* High system user, *n* Count, *Q*1 25th percentile, *Q*3 75th percentile, *SD* Standard deviation

^a^Population centre type depends on population size and census metropolitan area (CMA) and census agglomeration (CA) designation. Urban areas have ≥ 1,000 people with a population density of ≥ 400 persons per km2 and include the core (large urban area with ≥ 50,000 people for a CMA or ≥ 10,000 for a CA), secondary core (core merged with an adjacent CMA or larger CA), and population centres outside CMA/CAs. These three have been combined to be large urban areas. A fringe area is a small urban area within a CMA or CA that are not contiguous with the core of the CMA or CA classifications. A rural area is a region within a CMA or CA that is not core or fringe. https://www150.statcan.gc.ca/n1/pub/92-195-x/2011001/geo/rur/rur-eng.htm

^‡^Suppressed for data confidentiality reasons

### Community to ED transition

**Fig. 2:**
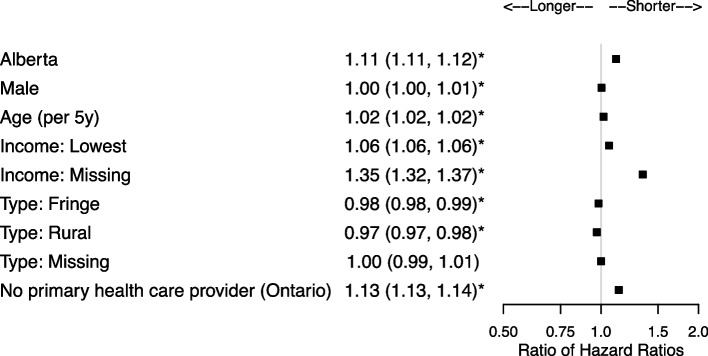
Ratio of HSU vs control hazard ratios and associated 95% confidence intervals for the community to ED transition. Reference categories are Ontario, female, combined medium–low to highest income quintile, large urban area, and family physician or other health care provider

Compared to Ontario, Alberta patients had shorter transition times for both HSUs (HR = 1.21, 95%CI 1.21, 1.22) and controls (HR = 1.09, 95%CI 1.09, 1.09; Supplementary Table 6, Additional File 1). HSUs in both provinces had even shorter community to ED transition times (HSU-to-control RHR = 1.11, 95%CI 1.11, 1.12; Fig. 2). In Ontario primary health care provider data were available and HSUs without a primary health care provider had shorter transition times than controls (RHR = 1.13, 95%CI 1.13, 1.14). Patient characteristics associated with shorter community to ED transition times for HSUs included living in areas in the lower income quintile (RHR = 1.06, 95%CI 1.06, 1.06) or where income was missing (RHR = 1.35, 95%CI 1.32, 1.37), and increasing age (RHR = 1.02 per 5 years, 95%CI 1.02, 1.02), and. HSUs had longer times if they resided in fringe (RHR = 0.98, 95%CI 0.98, 0.99) or rural areas (RHR = 0.97, 95%CI 0.97, 0.98) compared to patients in large urban areas.

### ED to death transition

There were 4,380 patients who died in the ED and fewer diagnosis categories were included in modeling because of small numbers. The median time from ED start to death was 57 min (IQR = 15 min, 3h37min). Characteristics associated with shorter ED to death transition times for HSUs were patients presenting with cancer (RHR = 2.51, 95%CI 1.93, 3.38; Supplementary Table 7, Supplementary Fig. 1, Additional File 1), general signs and symptoms (RHR = 2.31, 95%CI 1.59, 3.39), congestive heart failure (RHR = 1.93, 95%CI 1.25, 2.89), stroke (RHR = 1.84, 95% CI 1.26, 2.80), and myocardial infarction (RHR = 1.53, 95%CI 1.25, 1.84). Longer times were for patients presenting with peripheral vascular disease (RHR = 0.55, 95%CI 0.29, 0.79), Ontarians without a primary health care provider (RHR = 0.60; 95%CI 0.48, 0.74), males (RHR = 0.71, 95%CI 0.65, 0.79), Alberta residents (RHR = 0.86, 95%CI 0.76, 0.98), and younger age.

### ED to hospital transition

**Fig. 3:**
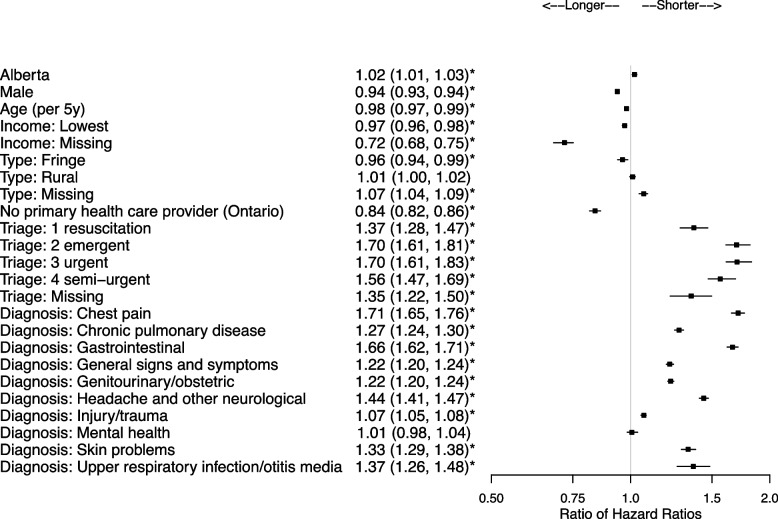
Ratio of HSU vs control hazard ratios and associated 95% confidence intervals for the ED to hospital transition (key diagnostic groups presented). Reference categories are Ontario, female, combined medium–low to highest income quintile, large urban area, family physician or other health care provider, CTAS 5 non-urgent, and diagnoses other than those presented in Supplementary Table 5, Additional File 1

Once at the ED, characteristics associated with shorter ED to hospital transition times for HSUs included higher acuity (Fig. 3, Supplementary Table 8, Additional File 1), and for most of the key diagnostic groups except mental health (Fig. 3, Supplementary Table 9, Additional File 1). Characteristics associated with longer ED to hospital transition times for HSUs included residence in an area where income was low or missing and a lack of primary health care provider. The median time from ED start to hospitalization was 6h13min (IQR = 3h53min, 9h27min).

### ED to community transition

**Fig. 4:**
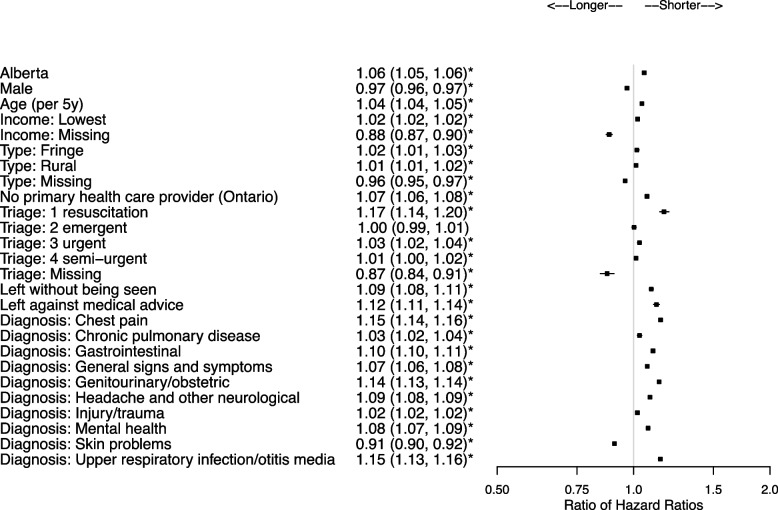
Ratio of HSU vs control hazard ratios and associated 95% confidence intervals for the ED to community transition (key diagnostic groups presented). Reference categories are Ontario, female, combined medium–low to highest income quintile, large urban area, family physician or other health care provider, CTAS 5 non-urgent, and diagnoses other than those presented in Supplementary Table 5, Additional File 1

Characteristics of patients associated with shorter ED to community transition times (Fig. 4, Supplementary Tables 8 and 9, Additional File 1) for HSUs included residence in Alberta, increasing age, residence in areas with lower income quintile, residence in fringe or rural areas, lack of a primary health care provider, being assigned CTAS 1, 3 or 4, and for most of the key diagnostic groups except skin problems. Transition times were also shorter for HSUs for patients who left without being seen (RHR = 1.09, 95%CI 1.08, 1.11) or left against medical advice (RHR = 1.12, 95%CI 1.11, 1.14). Characteristics associated with longer ED to community transition times for HSUs included male sex and from a missing income area. The median time from ED start to community was 2h44min (IQR = 1h29min, 4h50min).

### Hospital to death transition

There were 34,210 patients who died during their hospital admission and fewer diagnosis categories were included in modeling because of small numbers. The median time from hospitalization start to death was 6d20h50min (IQR = 2d9h16min, 16d17h31min). Characteristics associated with shorter hospital to death transition times for HSUs were patients presenting with cancer (RHR = 1.29, 95%CI 1.21, 1.38) and from rural areas (RHR = 1.07, 95%CI 1.02, 1.12; Supplementary Table 7, Supplementary Fig. 2, Additional File 1). Longer times were for patients presenting for some diagnose such as liver disease (RHR = 0.65, 95%CI 0.57, 0.73), headache or other neurological (RHR = 0.69, 95% 0.60, 0.79), and peptic ulcer disease (RHR = 0.69, 95%CI 0.65, 0.89). Times were also longer for patients who lacked a primary health care provider (RJR = 0.71, 95%CI 0.64, 0.80), resided in areas with lower incomes (RHR = 0.86, 95%CI 0.82, 0.88), and younger.

### Hospital to community transition

Characteristics associated with shorter hospital to community transition times (i.e., shorter hospital lengths of stay) for HSUs included residence in Alberta, male sex, increasing age, and residence in areas with lower or missing income quintile, and a lack of primary health care provider (Supplementary Fig. 3, Supplementary Table 8, Additional File 1). For diagnoses, ratios of HRs were mixed (Supplementary Fig. 3, Supplementary Table 9, Additional File 1) with mental health (RHR = 1.56, 95%CI 1.53, 1.60), chronic pulmonary disease, genitourinary/obstetric, headache and other neurological, and skin problems being associated with shorter hospital to community transition times for HSUs. Conversely, diagnoses associated with longer hospital to community transition times for HSUs included chest pain and general signs and symptoms. The median time from hospitalization start to community was 3d16h55min (IQR = 1d20h58min, 7d20h21min).

### Community to hospital transition

There were 152,813 transitions from community to hospital so, as expected, the vast majority of the patients in this study accessed hospital care via presentation to an ED. Characteristics associated with longer community to hospital transition times for HSUs included residence in areas with lower or missing income quintile and residence in Alberta (Supplementary Fig. 4, Additional File 1). Characteristics associated with shorter community to hospital transition times for HSUs included male sex (RHR = 1.62, 95%CI 1.59, 1.64), lack of a primary health care provider (RHR = 1.13, 95%CI 1.07, 1.19), and older ages. In addition, rural areas had shorter community to hospital transition times for HSUs. Estimates for the cumulative hazard function of this transition and the others are provided in Supplementary Fig. 5, Additional File 1.

## Discussion

This study used a large extract of HSUs and controls to evaluate transitions among four care states using multistate modeling. The advantage of examining what predictors relate to time patients spend in each state, compared to merely examining rates of interactions with services, is a better ability to predict patient flow within the health system.

Most transitions to hospital occurred through the ED, suggesting that acute exacerbations of chronic conditions—that may be sensitive to more optimal control in the community—were a driver of hospital use. The lack of a primary health care provider in Ontario led to longer times from community to ED for controls but shorter times for HSUs, suggesting a key role for primary care providers in managing HSUs’ health needs in the community. The fact that HSUs’ and controls’ times to transitions were differently impacted by their diagnoses supports the notion that disease-specific interventions may be useful in reducing the need for hospitalization among HSUs. As HSUs frequently have multiple co-morbidities [35], interventions could consider multi-morbidity in addition to accessibility to comprehensive primary care [36, 37].

Different impacts of some predictors (e.g. sex, age, income and geography) for HSUs and controls further suggests that serving the needs of HSUs will require specialized strategies targeted at their individual characteristics or underlying medical problems, rather than one size fits all solutions that may be adopted to serve “the average patient” in community, ED or hospital systems. Specifically, the finding that increasing age and lack of primary care predict both transitions from community to ED and from hospital to community suggest that interventions targeting older patients with decreased primary care access may reduce the frequency of health system use. Patient-specific interventions or targeted approaches may better meet HSUs health care needs and safely improve appropriate use of EDs. Variations on case management have been shown to be widely effective in reducing ED use among HSUs, although less is known about their clinical effectiveness, such that these approaches should be studied further [5].

The relatively few patients that transitioned directly from the community to hospital had shorter transition times if they were in Alberta, were female, and were older. It is not clear if these transitions represent planned admissions (e.g., for surgery or chemotherapy). Such planned admissions may be an efficient and patient-oriented process involving direct admission of HSUs from outpatient clinics. However, these transitions could also represent potentially unsafe bypass of ED (where diagnostic expertise is concentrated).

There are no directly comparable investigations of frequent ED users and controls in terms of transitions among ED, hospital, death, and community states. A few studies have looked at frequent ED users and their hospitalizations. In three Australian EDs, Berry et al. [38] focused on 115 seniors with frequent ED use and compared hospital admissions at the first and last ED presentations. Over time, they had more hospital stays > 7 days, suggesting deteriorating health status. Their study did not consider a control group or examine the times between events. In Korea among 156,246 ED users, 3.1% were frequent ED users and these users had longer stays when admitted to hospital [39]. Frequent ED users in Korea with type 2 diabetes (*n* = 849) were also shown to have longer stays when admitted to hospital compared to non-frequent ED users (*n* = 7,895) [40]. While these Korean studies did examine hospitalization length of stay, the timing of ED presentations and hospitalization events were not considered.

In the future, analyses could use smaller jurisdictions and include other aspects like ED characteristics and clinical data. Diagnosis groups, like patients with a specific chronic condition or combinations of multiple morbidities, may also provide new insights on the flow of patients through different care settings. Separately, future studies of patients’ ED use and outcomes, or of interventions related to ED use and outcomes, should account for frequency of ED use. The fact that the relationships between demographic factors, diseases and transitions timing differed between controls and HSUs means that failure to account for frequency of ED use will bias any studies results.

Our study limitations include the fact that administrative datasets are unable to provide granular clinical and treatment data, or data on housing status, family supports and substance use. Thus, we likely miss some important variables in the modeling because they are not collected as part of administrative processes. This study also did not include all health services used and for example, primary care physician visits could be another care state to add to a multistate model. Vital Statistics data were not available to allow for transitions from community to death to be included in the modeling. About 55% of deaths in the two provinces happen in hospital [41] and if deaths in this cohort are representative of the whole population, the 45% of deaths in the community would amount to a relatively small amount of 1.2% of our cohort that may have died that we are unable to capture.

## Conclusions

We used a multistate model to identify differential effects of predictors for HSUs and controls on transitions among four care settings. Co-morbidities and limitations in access to primary care are associated with more rapid transitions from community to ED and hospital among HSUs. Interventions targeting these challenges may better serve patients across health systems.

### Supplementary Information

**Additional file 1: Supplementary Table 1. **Diagnosis categories.** Supplementary Table 2. **Characteristics of HSUs and controls by provinces. **Supplementary Table 3. **Characteristics of Alberta patients and events at the different transitions. **Supplementary Table 4. **Characteristics of Ontario patients and events at the different transitions. **Supplementary Table 5. **Diagnoses at the ED to community, ED to hospital, hospital to community, ED to death, and hospital to death transitions.** Supplementary Table 6. **Hazard ratios (HRs) and associated 95% confidence intervals (CIs) for multivariable models for the transitions from community.** Supplementary Table 7. **Hazard ratios (HRs) and associated 95% confidence intervals (CIs) for multivariable models for the transitions to death.** Supplementary Table 8.** Hazard ratios (HRs) and associated 95% confidence intervals (CIs) for multivariable models for the transitions to ED to community, ED to hospital, and hospital to community: variables not related to diagnosis. **Supplementary Table 9. **Hazard ratios (HRs) and associated 95% confidence intervals (CIs) for multivariable models for the transitions to ED to community, ED to hospital, and hospital to community: variables related to diagnosis.** Supplementary Figure 1. **Ratio of HSU vs control hazard ratios and associated 95% confidence intervals for the ED to death transition. Reference categories are Ontario, female, combined medium-low to highest income quintile, large urban area, and family physician or other health care provider.** Supplementary Figure 2. **Ratio of HSU vs control hazard ratios and associated 95% confidence intervals for the hospital to death transition. Reference categories are Ontario, female, combined medium-low to highest income quintile, large urban area, and family physician or other health care provider.** Supplementary Figure 3. **Ratio of HSU vs control hazard ratios and associated 95% confidence intervals for the hospital to community transition (key diagnostic groups presented). Reference categories are Ontario, female, combined medium-low to highest income quintile, large urban area, family physician or other health care provider, and diagnoses other than those presented in Supplementary Table 9.** Supplementary Figure 4. **Ratio of HSU vs control hazard ratios and associated 95% confidence intervals for the community to hospital transition. Reference categories are Ontario, female, combined medium-low to highest income quintile, large urban area, and family physician or other health care provider. **Supplementary Figure 5. **Cummulative hazard function estimates for HSUs and controls (CON) for the different state transitions.

## Data Availability

Data is the property of the Canadian Institute for Health Information and the authors are not allowed to provide the data. Requests can be made for the same data from the Canadian Institute for Health Information for researchers who meet the criteria for access to confidential data. Researchers are welcome to inquire for further information at https://www.cihi.ca/en/make-a-data-request and select the “Access Data Inquiry Form” to make a data request.

## Abbreviations

CI: Confidence interval
CIHI: Canadian Institute for Health Information
CIHR: Canadian Institutes of Health Research
CTAS: Canadian Triage and Acuity Score
d: Day
ED: Emergency department
h: Hour
HR: Hazard ratio
HSU: High system user
ICD-10-CA: International Classification of Diseases and Related Health Problems, Tenth Revision, The Canadian Enhancement of ICD-10
IQR: Interquartile range
km: Kilometre
LAMA: Left against medical advice
LOS: Emergency department length of stay
LWBS: Left without being seen
min: Minute
n: Count
N.A.: Not applicable
NACRS: National Ambulatory Care Reporting System
PIA: Physician initial assessment
Q1: First quartile (25th percentile)
Q3: Third quartile (75th percentile)
SD: Standard deviation
US: United States
