# Supplementary material for: Transitions in health care settings for frequent and infrequent users of emergency departments: a population-based retrospective cohort study
Source: BMC Health Serv Res. 2023 Nov 14;23:1250. doi: 10.1186/s12913-023-10260-w (PMC10644485; doi:10.1186/s12913-023-10260-w)
Supplement: Supplementary file 1 — Additional file 1: Supplementary Table 1. Diagnosis categories. Supplementary Table 2. Characteristics of HSUs and controls by provinces. Supplementary Table 3. Characteristics of Alberta patients and events at the different transitions. Supplementary Table 4. Characteristics of Ontario patients and events at the different transitions. Supplementary Table 5. Diagnoses at the ED to community, ED to hospital, hospital to community, ED to death, and hospital to death transitions. Supplementary Table 6. Hazard ratios (HRs) and associated 95% confidence intervals (CIs) for multivariable models for the transitions from community. Supplementary Table 7. Hazard ratios (HRs) and associated 95% confidence intervals (CIs) for multivariable models for the transitions to death. Supplementary Table 8. Hazard ratios (HRs) and associated 95% confidence intervals (CIs) for multivariable models for the transitions to ED to community, ED to hospital, and hospital to community: variables not related to diagnosis. Supplementary Table 9. Hazard ratios (HRs) and associated 95% confidence intervals (CIs) for multivariable models for the transitions to ED to community, ED to hospital, and hospital to community: variables related to diagnosis. Supplementary Figure 1. Ratio of HSU vs control hazard ratios and associated 95% confidence intervals for the ED to death transition. Reference categories are Ontario, female, combined medium-low to highest income quintile, large urban area, and family physician or other health care provider. Supplementary Figure 2. Ratio of HSU vs control hazard ratios and associated 95% confidence intervals for the hospital to death transition. Reference categories are Ontario, female, combined medium-low to highest income quintile, large urban area, and family physician or other health care provider. Supplementary Figure 3. Ratio of HSU vs control hazard ratios and associated 95% confidence intervals for the hospital to community transition (key diagnostic groups pr [file 12913_2023_10260_MOESM1_ESM.docx]

**Additional File 1 for “Transitions in health care settings for frequent and infrequent users of emergency departments: a population-based retrospective cohort study”**

Supplementary Table 1. Diagnosis categories.

| **Category** | **ICD-10 Code** | **Source** |
| --- | --- | --- |
| AIDS/HIV | B20-B22, or B24 | Quan_deyo^1^ definitions from icd^2^ |
| Cancer | C00–C26, C30–C34, C37–C41, C43, C45–C58, C60–C76, C81-C85, C88, C90–C97 | Quan_deyo definitions from icd |
| Chest pain | R07 | Guttman et al.^3^ |
| Chronic pulmonary disease | I27.8, I27.9, J40–J47, J60–J67, J68.4, J70.1, or J70.3 | Quan_deyo definitions from icd |
| Congestive heart failure | I09.9, I11.0, I13.0, I13.2, I25.5, I42.0, I42.5–I42.9, I43, I50, or P29.0 | Quan_deyo definitions from icd |
| Dementia | F00–F03, F05.1, G30, or G31.1 | Quan_deyo definitions from icd |
| Diabetes | E10.0, E10.1, E10.6, E10.8, E10.9, E11.0, E11.1, E11.6, E11.8, E11.9, E12.0, E12.1, E12.6, E12.8, E12.9, E13.0, E13.1, E13.6, E13.8, E13.9, E14.0, E14.1, E14.6, E14.8, E14.9, E10.2–E10.5, E10.7, E11.2–E11.5, E11.7, E12.2–E12.5, E12.7, E13.2–E13.5, E13.7, E14.2–E14.5, or E14.7 | Quan_deyo definitions from icd |
| Fever | R50 | Guttman et al. |
| Gastrointestinal problems | R10, R11, or K52 | Guttman et al. |
| General signs and symptoms | K08, R53, R00, K62, R68, K59, K92, I10, or R60 | Guttman et al. |
| Genitourinary/obstetric excluding renal | R30, R31, R35, R33, N (not renal), or O | Guttman et al. |
| Headache and other neurological excluding stroke, dementia, paralysis | R51, R42, R55, R56, R41, H53, R40, R20, or G (not stroke, not dementia, not paralysis) | Guttman et al. |
| Hemiplegia or paraplegia | G04.1, G11.4, G80.1, G80.2, G81, G82, G83.0–G83.4, or G83.9 | Quan_deyo definitions from icd |
| Injury/trauma excluding rheumatic | Any ICD-10 codes starting with T, S, M (not rheumatic), or R04 | Guttman et al. |
| Liver | B18, K70.0–K70.3, K70.9, K71.3–K71.5, K71.7, K73, K74, K76.0, K76.2–K76.4, K76.8, K76.9, Z94.4, I85.0, I85.9, I86.4, I98.2, K70.4, K71.1, K72.1, K72.9, K76.5, K76.6, or K76.7 | Quan_deyo definitions from icd |
| Mental health excluding dementia | Any ICD-10 codes starting with F not dementia), or R45 | Guttman et al. |
| Metastatic solid tumour | C77–C80 | Quan_deyo definitions from icd |
| Myocardial infarction | I21-I22, or I25.2 | Quan_deyo definitions from icd |
| Peptic ulcer disease | K25–K28 | Quan_deyo definitions from icd |
| Peripheral vascular disease | I70, I71, I73.1, I73.8, I73.9, I77.1, I79.0, I79.2, K55.1, K55.8, K55.9, Z95.8, or Z95.9 | Quan_deyo definitions from icd |
| Renal | I12.0, I13.1, N03.2–N03.7, N05.2–N05.7, N18, N19, N25.0, Z49.0–Z49.2, Z94.0, or Z99.2 | Quan_deyo definitions from icd |
| Rheumatic | M05, M06, M31.5, M32–M34, M35.1, M35.3, or M36.0 | Quan_deyo definitions from icd |
| Shortness of breath | R06 | Guttman et al. |
| Skin problems | R21, R22, or L | Guttman et al. |
| Stroke | G45-G46, H34, I60-I69 | Quan_deyo definitions from icd |
| Upper Respiratory Tract Infections / Otitis media | R05, J02, H92, J00, J06, H66, or H10 | Guttman et al. |
| Other | Anything not already classified above |  |

^1^ Quan H, Sundararajan V, Halfon P, Fong A, Burnand B, Luthi J-C, Saunders LD, Beck CA,Feasby TE, Ghali WA (2005). Coding algorithms for defining comorbidities in ICD-9-CM and ICD-10 administrative data. Medical Care, 43(11), 1130-1139. http://www.ncbi.nlm.nih.gov/pubmed/16224307 <http://web.archive.org/web/20110225042437/http://www.chaps.ucalgary.ca/sas>^2^ Wasey JO, Lang M, R Core Team (2022). icd: Comorbidity Calculations and Tools for ICD-9 and ICD-10 Codes. R package version 4.0.9.9000. <https://jackwasey.github.io/icd/>

^3^ Guttmann A, Schull M J, Vermeulen M J, Stukel T A (2011). Association between waiting times and short term mortality and hospital admission after departure from emergency department: population based cohort study from Ontario, Canada. BMJ, 342:d2983 doi:10.1136/bmj.d2983

Supplementary Table 2. Characteristics of HSUs and controls by provinces.

|  |  | Alberta | | | | Ontario | | | |
| --- | --- | --- | --- | --- | --- | --- | --- | --- | --- |
|  |  | Control | | HSU | | Control | | HSU | |
| **Patient Characteristics** | | n=395,894 | | n=101,211 | | n=1,709,800 | | n=478,019 | |
| Sex, n (%) | |  |  |  |  |  |  |  |  |
|  | Female | 201,505 | (50.9) | 56,088 | (55.4) | 895,075 | (52.3) | 264,823 | (55.4) |
|  | Male | 194,389 | (49.1) | 45,123 | (44.6) | 814,725 | (47.7) | 213,196 | (44.6) |
| Age (years) | |  |  |  |  |  |  |  |  |
|  | Mean (SD) | 201,505 | (50.9) | 56,088 | (55.4) | 895,075 | (52.3) | 264,823 | (55.4) |
|  | Median (Q1, Q3) | 194,389 | (49.1) | 45,123 | (44.6) | 814,725 | (47.7) | 213,196 | (44.6) |
| Neighborhood Income Quintile, n (%) | |  |  |  |  |  |  |  |  |
|  | Lowest | 91,106 | (23.0) | 29,771 | (29.4) | 355,382 | (20.8) | 130,313 | (27.3) |
|  | Medium-low | 85,979 | (21.7) | 23,256 | (23.0) | 344,840 | (20.2) | 102,968 | (21.5) |
|  | Middle | 77,526 | (19.6) | 18,630 | (18.4) | 350,175 | (20.5) | 93,047 | (19.5) |
|  | Medium-high | 72,777 | (18.4) | 16,154 | (16.0) | 328,927 | (19.2) | 80,473 | (16.8) |
|  | Highest | 65,118 | (16.4) | 11,879 | (11.7) | 311,258 | (18.2) | 65,861 | (13.8) |
|  | Missing / Not applicable | 3,388 | (0.9) | 1,521 | (1.5) | 19,218 | (1.1) | 5,357 | (1.1) |
| Population Type†, n (%) | |  |  |  |  |  |  |  |  |
|  | Large urban | 307,985 | (77.8) | 64,781 | (64.0) | 1,302,267 | (76.2) | 340,666 | (71.3) |
|  | Fringe | 8,698 | (2.2) | 2,277 | (2.2) | 49,421 | (2.9) | 13,355 | (2.8) |
|  | Rural area | 21,240 | (5.4) | 7,002 | (6.9) | 301,717 | (17.6) | 102,002 | (21.3) |
|  | Missing (not linked to geographic level or not applicable) | 57,971 | (14.6) | 27,151 | (26.8) | 56,395 | (3.3) | 21,996 | (4.6) |
| Primary Health Care (Ontario only), n (%) | |  |  |  |  |  |  |  |  |
|  | Family Physician |  |  |  |  | 1,514,496 | (88.6) | 432,538 | (90.5) |
|  | None |  |  |  |  | 126,161 | (7.4) | 33,534 | (7.0) |
|  | Other (ex. Family Health Team, Walk-in Clinic) |  |  |  |  | 11,110 | (0.6) | 4,231 | (0.9) |
|  | Unknown/Unavailable/ Missing (includes cases where patient is unconscious or arrives dead) |  |  |  |  | 58,033 | (3.4) | 7,716 | (1.6) |
| Died, n (%) | |  |  |  |  |  |  |  |  |
|  | In-ED | 532 | (0.1) | 202 | (0.2) | 2,564 | (0.1) | 1,082 | (0.2) |
|  | In-hospital | 3,213 | (0.8) | 2,286 | (2.3) | 15,187 | (0.9) | 15,187 | (3.2) |

Supplementary Table 3. Characteristics of Alberta patients and events at the different transitions.

|  |  | Community to ED  n=1,045,504 | | ED to Community  n=927,803 | | ED to Hospital  n=116,967 | | Hospital to Community  n= 145,434 | | Community to Hospital  n=33,966 | | ED to Death  n=734 | | Hospital to Death  n=5,499 | |
| --- | --- | --- | --- | --- | --- | --- | --- | --- | --- | --- | --- | --- | --- | --- | --- |
| Distinct patients, n | | 496,746 | | 465,548 | | 82,649 | | 104,936 | | 32,350 | | 734 | | 5,499 | |
| Group, n (%) | |  |  |  |  |  |  |  |  |  |  |  |  |  |  |
|  | Control | 521,468 | (49.9) | 469,250 | (50.6) | 51,686 | (44.2) | 70,282 | (48.3) | 21,809 | (64.2) | 532 | (72.5) | 3,213 | (58.4) |
|  | HSU | 524,036 | (50.1) | 458,553 | (49.4) | 65,281 | (55.8) | 75,152 | (51.7) | 12,157 | (35.8) | 202 | (27.5) | 2,286 | (41.6) |
| Sex, n (%) | |  |  |  |  |  |  |  |  |  |  |  |  |  |  |
|  | Female | 558,080 | (53.4) | 498,769 | (53.8) | 59,055 | (50.5) | 79,507 | (54.7) | 22,973 | (67.6) | 256 | (34.9) | 2,521 | (45.8) |
|  | Male | 487,424 | (46.6) | 429,034 | (46.2) | 57,912 | (49.5) | 65,927 | (45.3) | 10,993 | (32.4) | 478 | (65.1) | 2,978 | (54.2) |
| Age (years) | |  |  |  |  |  |  |  |  |  |  |  |  |  |  |
|  | Mean (SD) | 47.4 | (19.9) | 45.8 | (19.2) | 59.4 | (21.0) | 56.3 | (21.1) | 48.5 | (19.7) | 66.1 | (18.0) | 74.1 | (14.9) |
|  | Median (Q1, Q3) | 45.0 | (30.0,62.0) | 43.0 | (30.0,59.0) | 61.0 | (43.0,77.0) | 58.0 | (37.0,74.0) | 46.0 | (31.0,65.0) | 68.0 | (55.0,80.8) | 77.0 | (65.0,85.0) |
| Neighborhood Income Quintile, n (%) | |  |  |  |  |  |  |  |  |  |  |  |  |  |  |
|  | Lowest | 286,671 | (27.4) | 251,023 | (27.1) | 35,458 | (30.3) | 42,182 | (29.0) | 8,391 | (24.7) | 190 | (25.9) | 1,667 | (30.3) |
|  | Medium-low | 232,356 | (22.2) | 205,824 | (22.2) | 26,366 | (22.5) | 32,487 | (22.3) | 7,442 | (21.9) | 166 | (22.6) | 1,321 | (24.0) |
|  | Middle | 195,781 | (18.7) | 174,844 | (18.8) | 20,796 | (17.8) | 26,427 | (18.2) | 6,593 | (19.4) | 141 | (19.2) | 962 | (17.5) |
|  | Medium-high | 173,479 | (16.6) | 155,671 | (16.8) | 17,695 | (15.1) | 22,950 | (15.8) | 6,092 | (17.9) | 113 | (15.4) | 837 | (15.2) |
|  | Highest | 141,115 | (13.5) | 126,321 | (13.6) | 14,687 | (12.6) | 19,252 | (13.2) | 5,246 | (15.4) | 107 | (14.6) | 681 | (12.4) |
|  | Missing / Not applicable | 16,102 | (1.5) | 14,120 | (1.5) | 1,965 | (1.7) | 2,136 | (1.5) | 202 | (0.6) | 17 | (2.3) | 31 | (0.6) |
| Population Type†, n (%) | |  |  |  |  |  |  |  |  |  |  |  |  |  |  |
|  | Large urban | 730,394 | (69.9) | 642,534 | (69.3) | 87,304 | (74.6) | 107,941 | (74.2) | 24,812 | (73.0) | 556 | (75.7) | 4,175 | (75.9) |
|  | Fringe | 23,601 | (2.3) | 21,499 | (2.3) | 2,080 | (1.8) | 2,738 | (1.9) | 786 | (2.3) | 22 | (3.0) | 128 | (2.3) |
|  | Rural area | 63,542 | (6.1) | 57,422 | (6.2) | 6,085 | (5.2) | 7,857 | (5.4) | 2,056 | (6.1) | 35 | (4.8) | 284 | (5.2) |
|  | Missing (not linked to geographic level or not applicable) | 227,967 | (21.8) | 206,348 | (22.2) | 21,498 | (18.4) | 26,898 | (18.5) | 6,312 | (18.6) | 121 | (16.5) | 912 | (16.6) |
| Triage Level, n (%) | |  |  |  |  |  |  |  |  |  |  |  |  |  |  |
|  | 1- Resuscitation |  |  | 1,736 | (0.2) | 3,356 | (2.9) |  |  |  |  | 556 | (75.7) |  |  |
|  | 2-Emergent |  |  | 102,043 | (11.0) | 39,738 | (34.0) |  |  |  |  | 87 | (11.9) |  |  |
|  | 3-Urgent |  |  | 323,750 | (34.9) | 56,770 | (48.5) |  |  |  |  | 41 | (5.6) |  |  |
|  | 4-Semi-urgent |  |  | 360,100 | (38.8) | 13,065 | (11.2) |  |  |  |  | 14 | (1.9) |  |  |
|  | 5-Non-urgent |  |  | 115,815 | (12.5) | 1,465 | (1.3) |  |  |  |  | <10‡ |  |  |  |
|  | Missing/unavailable |  |  | 24,359 | (2.6) | 2,573 | (2.2) |  |  |  |  | 29 | (4.0) |  |  |

HSU=high system user; n=count; Q1=25th percentile; Q3=75th percentile; SD=standard deviation

† Population centre type depends on population size and census metropolitan area (CMA) and census agglomeration (CA) designation. Urban areas have ≥1,000 people with a population density of ≥400 persons per km2 and include the core (large urban area with ≥50,000 people for a CMA or ≥10,000 for a CA), secondary core (core merged with an adjacent CMA or larger CA), and population centres outside CMA/CAs. These three have been combined to be large urban areas. A fringe area is a small urban area within a CMA or CA that are not contiguous with the core of the CMA or CA classifications. A rural area is a region within a CMA or CA that is not core or fringe. <https://www150.statcan.gc.ca/n1/pub/92-195-x/2011001/geo/rur/rur-eng.htm>

‡ Suppressed for data confidentiality reasons.

Supplementary Table 4. Characteristics of Ontario patients and events at the different transitions.

|  |  | Community to ED  n=3,977,032 | | ED to Community  n=3,518,797 | | ED to Hospital  n=454,589 | | Hospital to Community  n=544,725 | | Community to Hospital  n=118,847 | | ED to Death  n=3,646 | | Hospital to Death  n=28,711 | |
| --- | --- | --- | --- | --- | --- | --- | --- | --- | --- | --- | --- | --- | --- | --- | --- |
| Distinct patients, n | | 2,186,970 | | 2,044,209 | | 327,762 | | 401,037 | | 114,249 | | 3,646 | | 28,711 | |
| Group, n (%) | |  |  |  |  |  |  |  |  |  |  |  |  |  |  |
|  | Control | 2,040,064 | (51.3) | 1,849,315 | (52.6) | 188,185 | (41.4) | 247,109 | (45.4) | 74,111 | (62.4) | 2,564 | (70.3) | 15,187 | (52.9) |
|  | HSU | 1,936,968 | (48.7) | 1,669,482 | (47.4) | 266,404 | (58.6) | 297,616 | (54.6) | 44,736 | (37.6) | 1,082 | (29.7) | 13,524 | (47.1) |
| Sex, n (%) | |  |  |  |  |  |  |  |  |  |  |  |  |  |  |
|  | Female | 2,142,987 | (53.9) | 1,909,498 | (54.3) | 232,160 | (51.1) | 294,791 | (54.1) | 76,163 | (64.1) | 1,329 | (36.5) | 13,532 | (47.1) |
|  | Male | 1,834,045 | (46.1) | 1,609,299 | (45.7) | 222,429 | (48.9) | 249,934 | (45.9) | 42,684 | (35.9) | 2,317 | (63.5) | 15,179 | (52.9) |
| Age (years) | |  |  |  |  |  |  |  |  |  |  |  |  |  |  |
|  | Mean (SD) | 50.8 | (20.7) | 48.8 | (20.0) | 65.8 | (19.3) | 62.4 | (20.1) | 52.9 | (19.9) | 69.4 | (17.3) | 75.9 | (13.8) |
|  | Median (Q1, Q3) | 50.0 | (33.0,67.0) | 48.0 | (31.0,64.0) | 69.0 | (54.0,81.0) | 66.0 | (49.0,79.0) | 55.0 | (34.0,69.0) | 72.0 | (59.0,83.0) | 78.0 | (68.0,86.0) |
| Neighborhood Income Quintile, n (%) | |  |  |  |  |  |  |  |  |  |  |  |  |  |  |
|  | Lowest | 999,417 | (25.1) | 878,948 | (25.0) | 119,550 | (26.3) | 138,486 | (25.4) | 26,500 | (22.3) | 919 | (25.2) | 7,564 | (26.3) |
|  | Medium-low | 828,759 | (20.8) | 731,302 | (20.8) | 96,704 | (21.3) | 114,705 | (21.1) | 24,393 | (20.5) | 753 | (20.7) | 6,392 | (22.3) |
|  | Middle | 779,479 | (19.6) | 691,243 | (19.6) | 87,497 | (19.2) | 106,340 | (19.5) | 24,340 | (20.5) | 739 | (20.3) | 5,497 | (19.1) |
|  | Medium-high | 700,989 | (17.6) | 624,923 | (17.8) | 75,458 | (16.6) | 93,444 | (17.2) | 22,685 | (19.1) | 608 | (16.7) | 4,699 | (16.4) |
|  | Highest | 618,676 | (15.6) | 547,682 | (15.6) | 70,437 | (15.5) | 86,226 | (15.8) | 20,053 | (16.9) | 557 | (15.3) | 4,264 | (14.9) |
|  | Missing / Not applicable | 49,712 | (1.2) | 44,699 | (1.3) | 4,943 | (1.1) | 5,524 | (1.0) | 876 | (0.7) | 70 | (1.9) | 295 | (1.0) |
| Population Type†, n (%) | |  |  |  |  |  |  |  |  |  |  |  |  |  |  |
|  | Large urban | 2,932,963 | (73.7) | 2,577,999 | (73.3) | 352,292 | (77.5) | 418,096 | (76.8) | 87,811 | (73.9) | 2,672 | (73.3) | 22,007 | (76.7) |
|  | Fringe | 110,607 | (2.8) | 98,366 | (2.8) | 12,112 | (2.7) | 14,874 | (2.7) | 3,505 | (2.9) | 129 | (3.5) | 743 | (2.6) |
|  | Rural area | 766,979 | (19.3) | 693,418 | (19.7) | 72,883 | (16.0) | 91,331 | (16.8) | 23,306 | (19.6) | 678 | (18.6) | 4,858 | (16.9) |
|  | Missing (not linked to geographic level or not applicable) | 166,483 | (4.2) | 149,014 | (4.2) | 17,302 | (3.8) | 20,424 | (3.7) | 4,225 | (3.6) | 167 | (4.6) | 1,103 | (3.8) |
| Primary Health Care, n (%) | |  |  |  |  |  |  |  |  |  |  |  |  |  |  |
|  | Family Physician | 3,561,846 | (89.6) | 3,131,906 | (89.0) | 426,819 | (93.9) | 511,776 | (94.0) | 112,100 | (94.3) | 3,121 | (85.6) | 27,143 | (94.5) |
|  | None | 293,541 | (7.4) | 275,383 | (7.8) | 17,918 | (3.9) | 21,192 | (3.9) | 4,156 | (3.5) | 240 | (6.6) | 882 | (3.1) |
|  | Other (ex. Family Health Team, Walk-in Clinic) | 31,777 | (0.8) | 29,378 | (0.8) | 2,382 | (0.5) | 3,023 | (0.6) | 774 | (0.7) | 17 | (0.5) | 133 | (0.5) |
|  | Unknown/Unavailable/ Missing (includes cases where patient is unconscious or arrives dead) | 89,868 | (2.3) | 82,130 | (2.3) | 7,470 | (1.6) | 8,734 | (1.6) | 1,817 | (1.5) | 268 | (7.4) | 553 | (1.9) |
| Triage Level, n (%) | |  |  |  |  |  |  |  |  |  |  |  |  |  |  |
|  | 1- Resuscitation |  |  | 10,355 | (0.3) | 23,904 | (5.3) |  |  |  |  | 2,888 | (79.2) |  |  |
|  | 2-Emergent |  |  | 601,841 | (17.1) | 209,573 | (46.1) |  |  |  |  | 527 | (14.5) |  |  |
|  | 3-Urgent |  |  | 1,611,428 | (45.8) | 200,744 | (44.2) |  |  |  |  | 158 | (4.3) |  |  |
|  | 4-Semi-urgent |  |  | 1,126,254 | (32.0) | 18,268 | (4.0) |  |  |  |  | 20 | (0.5) |  |  |
|  | 5-Non-urgent |  |  | 151,361 | (4.3) | 1,379 | (0.3) |  |  |  |  | 38 | (1.0) |  |  |
|  | Missing/unavailable |  |  | 17,558 | (0.5) | 721 | (0.2) |  |  |  |  | 15 | (0.4) |  |  |

HSU=high system user; n=count; Q1=25th percentile; Q3=75th percentile; SD=standard deviation

† Population centre type depends on population size and census metropolitan area (CMA) and census agglomeration (CA) designation. Urban areas have ≥1,000 people with a population density of ≥400 persons per km2 and include the core (large urban area with ≥50,000 people for a CMA or ≥10,000 for a CA), secondary core (core merged with an adjacent CMA or larger CA), and population centres outside CMA/CAs. These three have been combined to be large urban areas. A fringe area is a small urban area within a CMA or CA that are not contiguous with the core of the CMA or CA classifications. A rural area is a region within a CMA or CA that is not core or fringe. <https://www150.statcan.gc.ca/n1/pub/92-195-x/2011001/geo/rur/rur-eng.htm>

‡ Suppressed for data confidentiality reasons.

Supplementary Table 5. Diagnoses at the ED to community, ED to hospital, hospital to community, ED to death, and hospital to death transitions.

|  | ED to Community  n=2,509,757  n (%) | | ED to Hospital  n=571,556  n (%) | | Hospital to Community† n=690,159  n (%) | | ED to Death  n=4,380  n (%) | | Hospital to Death†  n=34,210  n (%) | |
| --- | --- | --- | --- | --- | --- | --- | --- | --- | --- | --- |
| Distinct patients | 2,509,757 | | 410,411 | | 505,973 | | 4,380 | | 34,210 | |
| AIDS/HIV | 113 | (0.0) | 147 | (0.0) | 331 | (0.0) | <10‡ |  | 17 | (0.0) |
| Cancer | 7,800 | (0.2) | 7,917 | (1.4) | 29,350 | (4.3) | 92 | (2.1) | 2,596 | (7.6) |
| Chest pain | 201,258 | (4.5) | 8,068 | (1.4) | 7,129 | (1.0) | <10 |  | <10 |  |
| Chronic pulmonary disease | 92,332 | (2.1) | 24,826 | (4.3) | 28,510 | (4.1) | 31 | (0.7) | 1,346 | (3.9) |
| Congestive heart failure | 12,411 | (0.3) | 21,341 | (3.7) | 22,768 | (3.3) | 50 | (1.1) | 1,893 | (5.5) |
| Dementia | 3,600 | (0.1) | 2,120 | (0.4) | 4,947 | (0.7) | <10 |  | 324 | (0.9) |
| Diabetes | 22,601 | (0.5) | 10,045 | (1.8) | 12,131 | (1.8) | 12 | (0.3) | 280 | (0.8) |
| Fever | 11,953 | (0.3) | 5,784 | (1.0) | 1,996 | (0.3) | 0 | (0.0) | 10 | (0.0) |
| Gastrointestinal | 239,897 | (5.4) | 17,354 | (3.0) | 9,287 | (1.3) | 10 | (0.2) | 69 | (0.2) |
| General signs and symptoms | 163,086 | (3.7) | 30,140 | (5.3) | 16,046 | (2.3) | 64 | (1.5) | 382 | (1.1) |
| Genitourinary/obstetric | 363,393 | (8.2) | 45,561 | (8.0) | 99,364 | (14.4) | 23 | (0.5) | 1,025 | (3.0) |
| Headache and other neurological | 240,819 | (5.4) | 28,403 | (5.0) | 22,181 | (3.2) | 33 | (0.8) | 639 | (1.9) |
| Hemiplegia or paraplegia | 312 | (0.0) | 292 | (0.1) | 193 | (0.0) | 0 | (0.0) | <10 |  |
| Injury/trauma | 1,383,459 | (31.1) | 77,598 | (13.6) | 103,912 | (15.1) | 282 | (6.4) | 1,935 | (5.7) |
| Liver | 2,412 | (0.1) | 3,661 | (0.6) | 4,763 | (0.7) | 16 | (0.4) | 709 | (2.1) |
| Mental health | 219,522 | (4.9) | 19,391 | (3.4) | 22,063 | (3.2) | <10 |  | 154 | (0.5) |
| Metastatic solid tumour | 2,046 | (0.0) | 2,541 | (0.4) | 6,131 | (0.9) | <10 |  | 831 | (2.4) |
| Myocardial infarction | 453 | (0.0) | 6,261 | (1.1) | 17,605 | (2.6) | 344 | (7.9) | 1,225 | (3.6) |
| Peptic ulcer disease | 2,550 | (0.1) | 734 | (0.1) | 3,673 | (0.5) | <10 |  | 136 | (0.4) |
| Peripheral vascular disease | 1,938 | (0.0) | 1,784 | (0.3) | 3,813 | (0.6) | 81 | (1.8) | 338 | (1.0) |
| Renal disease | 1,502 | (0.0) | 1,261 | (0.2) | 949 | (0.1) | <10 |  | 83 | (0.2) |
| Rheumatic disease | 2,365 | (0.1) | 425 | (0.1) | 924 | (0.1) | 0 | (0.0) | 16 | (0.0) |
| Shortness of breath | 29,629 | (0.7) | 6,284 | (1.1) | 1,378 | (0.2) | 14 | (0.3) | 35 | (0.1) |
| Skin problems | 207,274 | (4.7) | 9,628 | (1.7) | 9,490 | (1.4) | <10 |  | 112 | (0.3) |
| Stroke | 17,702 | (0.4) | 20,292 | (3.6) | 18,231 | (2.6) | 103 | (2.4) | 1,967 | (5.7) |
| Upper respiratory infections | 188,956 | (4.2) | 1,144 | (0.2) | 1,121 | (0.2) | <10 |  | <10 |  |
| Others | 1,027,217 | (23.1) | 218,554 | (38.2) | 241,873 | (35.0) | 3,190 | (72.8) | 18,073 | (52.8) |

n=number of transitions; †=hospital diagnosis; ‡ Suppressed for data confidentiality reasons.

Supplementary Table 6. Hazard ratios (HRs) and associated 95% confidence intervals (CIs) for multivariable models for the transitions from community.

|  |  | Community to ED | | | | Community to Hospital | | | |
| --- | --- | --- | --- | --- | --- | --- | --- | --- | --- |
|  | | Control | | HSU | | Control | | HSU | |
|  | | HR | (95% CI) | HR | (95% CI) | HR | (95% CI) | HR | (95% CI) |
| Province (reference=Ontario) | |  |  |  |  |  |  |  |  |
|  | Alberta | 1.09 | (1.09, 1.09)* | 1.21 | (1.21, 1.22)* | 1.23 | (1.22, 1.25)* | 1.21 | (1.19, 1.23)* |
| Sex (reference=female) | |  |  |  |  |  |  |  |  |
|  | Male | 1.01 | (1.01, 1.02)* | 1.02 | (1.02, 1.02)* | 0.53 | (0.53, 0.54)* | 0.86 | (0.85, 0.87)* |
| Per 5 years of age | | 1.01 | (1.01, 1.02)* | 1.03 | (1.03, 1.04)* | 1.11 | (1.11, 1.12)* | 1.18 | (1.17, 1.19)* |
| Income (reference=higher quintile) | |  |  |  |  |  |  |  |  |
|  | Lowest quintile | 1.02 | (1.02, 1.02)* | 1.08 | (1.07, 1.08)* | 0.96 | (0.95, 0.97)* | 0.91 | (0.90, 0.92)* |
|  | Missing quintile | 0.95 | (0.94, 0.96)* | 1.28 | (1.27, 1.30)* | 0.78 | (0.74, 0.82)* | 0.53 | (0.49, 0.57)* |
| Population Type (reference=large urban) | |  |  |  |  |  |  |  |  |
|  | Fringe | 1.00 | (1.00, 1.01) | 0.98 | (0.98, 0.99)* | 1.03 | (1.00, 1.06) | 1.03 | (0.99, 1.08) |
|  | Rural area | 1.02 | (1.02, 1.02)* | 0.99 | (0.99, 1.00)* | 1.00 | (0.99, 1.02) | 1.07 | (1.05, 1.09)* |
|  | Missing area | 1.03 | (1.03, 1.04)* | 1.03 | (1.03, 1.04)* | 1.00 | (0.98, 1.02) | 1.05 | (1.02, 1.07)* |
| Primary Health Care Provider (Ontario only, reference= family physician, other, missing, unknown/ unavailable) | |  |  |  |  |  |  |  |  |
|  | None | 0.95 | (0.95, 0.96)* | 1.08 | (1.07, 1.09)* | 0.51 | (0.49, 0.52)* | 0.58 | (0.55, 0.60)* |

Supplementary Table 7. Hazard ratios (HRs) and associated 95% confidence intervals (CIs) for multivariable models for the transitions to death.

|  |  | ED to Death | | | | Hospital to Death† | | | |
| --- | --- | --- | --- | --- | --- | --- | --- | --- | --- |
|  | | Control | | HSU | | Control | | HSU | |
|  | | HR | (95% CI) | HR | (95% CI) | HR | (95% CI) | HR | (95% CI) |
| Province (reference=Ontario) | |  |  |  |  |  |  |  |  |
|  | Alberta | 0.95 | (0.89, 1.02) | 0.81 | (0.73, 0.89)* | 0.70 | (0.68, 0.72)* | 0.68 | (0.66, 0.71)* |
| Sex (reference=female) | |  |  |  |  |  |  |  |  |
|  | Male | 2.26 | (2.13, 2.39)* | 1.60 | (1.49, 1.74)* | 1.16 | (1.14, 1.19)* | 1.20 | (1.17, 1.23)* |
| Per 5 years of age | | 3.19 | (3.05, 3.33)* | 2.72 | (2.58, 2.86)* | 2.53 | (2.47, 2.57)* | 1.95 | (1.92, 1.99)* |
| Income (reference=higher quintile) | |  |  |  |  |  |  |  |  |
|  | Lowest quintile | 1.11 | (1.04, 1.18)* | 1.06 | (0.96, 1.17) | 1.12 | (1.09, 1.15)* | 0.96 | (0.93, 0.99)* |
|  | Missing quintile | 2.54 | (2.04, 3.13)* | 0.84 | (0.54, 1.26) | 0.99 | (0.85, 1.18) | 0.87 | (0.77, 0.95)* |
| Population Type (reference=large urban) | |  |  |  |  |  |  |  |  |
|  | Fringe | 1.20 | (1.00, 1.35) | 1.52 | (1.24, 1.81)* | 1.01 | (0.94, 1.09) | 1.04 | (0.96, 1.12) |
|  | Rural area | 1.13 | (1.05, 1.22)* | 1.02 | (0.92, 1.14) | 1.04 | (1.01, 1.08)* | 1.11 | (1.08, 1.15)* |
|  | Missing area | 0.99 | (0.82, 1.12) | 1.05 | (0.86, 1.23) | 1.10 | (1.05, 1.18)* | 1.14 | (1.09, 1.19)* |
| Primary Health Care Provider(Ontario only, reference= family physician, other, missing, unknown/ unavailable) | |  |  |  |  |  |  |  |  |
|  | None | 1.81 | (1.58, 1.98)* | 1.09 | (0.91, 1.29) | 1.23 | (1.16, 1.30)* | 0.87 | (0.80, 0.96)* |
| Diagnosis (reference=all other diagnoses) | |  |  |  |  |  |  |  |  |
|  | Cancer | 1.73 | (1.36, 2.16)* | 4.35 | (3.49, 5.28)* | 0.77 | (0.73, 0.80)* | 0.99 | (0.94, 1.02) |
|  | Congestive heart failure | 0.37 | (0.27, 0.48)* | 0.71 | (0.54, 0.90)* | 0.98 | (0.93, 1.03) | 0.89 | (0.84, 0.93)* |
|  | Diabetes |  |  |  |  | 0.47 | (0.39, 0.52)* | 0.38 | (0.33, 0.44)* |
|  | General signs and symptoms | 0.14 | (0.10, 0.18)* | 0.33 | (0.24, 0.42)* | 0.49 | (0.44, 0.54)* | 0.42 | (0.37, 0.47)* |
|  | Genitourinary/ obstetric | 0.05 | (0.03, 0.07)* | 0.05 | (0.02, 0.08)* | 0.36 | (0.34, 0.38)* | 0.37 | (0.35, 0.39)* |
|  | Headache and other neurological |  |  |  |  | 0.68 | (0.63, 0.73)* | 0.47 | (0.42, 0.50)* |
|  | Injury/trauma | 0.17 | (0.16, 0.19)* | 0.20 | (0.16, 0.23)* | 0.29 | (0.27, 0.30)* | 0.30 | (0.28, 0.31)* |
|  | Liver | 2.10 | (1.13, 2.98)* | 1.57 | (0.73, 2.59) | 3.22 | (2.95, 3.47)* | 2.08 | (1.93, 2.27)* |
|  | Myocardial infarction | 16.97 | (15.45, 18.24)* | 26.05 | (22.22, 30.21)* | 1.46 | (1.40, 1.54)* | 1.07 | (0.99, 1.15) |
|  | Peptic ulcer disease |  |  |  |  | 0.79 | (0.69, 0.90)* | 0.54 | (0.42, 0.67)* |
|  | Peripheral vascular disease | 8.16 | (6.76, 9.55)* | 4.46 | (2.27, 6.31)* | 0.98 | (0.86, 1.08) | 0.75 | (0.65, 0.87)* |
|  | Renal disease |  |  |  |  | 0.88 | (0.61, 1.14) | 1.04 | (0.83, 1.28) |
|  | Shortness of breath | 0.28 | (0.16, 0.41)* | 0.09 | (0.00, 0.24)* | 0.86 | (0.61, 1.13) | 0.53 | (0.31, 0.73)* |
|  | Stroke | 0.74 | (0.58, 0.87)* | 1.37 | (1.00, 1.75) | 1.31 | (1.26, 1.36)* | 0.99 | (0.93, 1.04) |

†=hospital diagnosis

Supplementary Table 8. Hazard ratios (HRs) and associated 95% confidence intervals (CIs) for multivariable models for the transitions to ED to community, ED to hospital, and hospital to community: variables not related to diagnosis.

|  |  | ED to Community | | | | ED to Hospital | | | | Hospital to Community | | | | |
| --- | --- | --- | --- | --- | --- | --- | --- | --- | --- | --- | --- | --- | --- | --- |
|  | | Control | | HSU | | Control | | HSU | | Control | | | HSU | |
|  | | HR | (95% CI) | HR | (95% CI) | HR | (95% CI) | HR | (95% CI) | | HR | (95% CI) | HR | (95% CI) |
| Province (reference=Ontario) | |  |  |  |  |  |  |  |  | |  |  |  |  |
|  | Alberta | 0.89 | (0.89, 0.90)* | 0.94 | (0.94, 0.95)* | 1.2 | (1.19, 1.21)* | 1.22 | (1.21, 1.23)* | | 0.89 | (0.89, 0.90)* | 0.91 | (0.91, 0.92)* |
| Sex (reference=female) | |  |  |  |  |  |  |  |  | |  |  |  |  |
|  | Male | 1.01 | (1.01, 1.01)* | 0.98 | (0.97, 0.98)* | 1.14 | (1.13, 1.15)* | 1.07 | (1.06, 1.07)* | | 0.95 | (0.94, 0.95)* | 0.99 | (0.99, 1.00)* |
| Per 5 years of age | | 0.81 | (0.81, 0.81)* | 0.84 | (0.84, 0.84)* | 1.58 | (1.57, 1.59)* | 1.55 | (1.54, 1.56)* | | 0.66 | (0.66, 0.66)* | 0.76 | (0.75, 0.76)* |
| Income (reference=higher quintile) | |  |  |  |  |  |  |  |  | |  |  |  |  |
|  | Lowest quintile | 0.97 | (0.97, 0.98)* | 0.99 | (0.99, 1.00)* | 1.05 | (1.04, 1.06)* | 1.02 | (1.01, 1.03)* | | 0.93 | (0.93, 0.94)* | 0.97 | (0.97, 0.98)* |
|  | Missing quintile | 0.80 | (0.80, 0.81)* | 0.71 | (0.70, 0.72)* | 0.88 | (0.85, 0.91)* | 0.64 | (0.61, 0.65)* | | 0.84 | (0.81, 0.88)* | 0.94 | (0.92, 0.97)* |
| Population Type (reference=large urban) | |  |  |  |  |  |  |  |  | |  |  |  |  |
|  | Fringe | 1.06 | (1.05, 1.07)* | 1.08 | (1.07, 1.09)* | 1.00 | (0.97, 1.01) | 0.96 | (0.94, 0.97)* | | 0.99 | (0.97, 1.01) | 1.00 | (0.98, 1.01) |
|  | Rural area | 1.19 | (1.18, 1.19)* | 1.20 | (1.20, 1.21)* | 1.18 | (1.17, 1.19)* | 1.19 | (1.18, 1.20)* | | 1.04 | (1.03, 1.05)* | 1.02 | (1.01, 1.02)* |
|  | Missing area | 1.23 | (1.22, 1.23)* | 1.18 | (1.17, 1.18)* | 1.23 | (1.21, 1.25)* | 1.31 | (1.29, 1.33)* | | 1.05 | (1.03, 1.07)* | 1.04 | (1.03, 1.06)* |
| Primary Health Care Provider (Ontario only, reference= family physician, other, missing, unknown/ unavailable) | |  |  |  |  |  |  |  |  | |  |  |  |  |
|  | None | 0.99 | (0.98, 0.99)* | 1.06 | (1.05, 1.06)* | 0.98 | (0.96, 0.99)* | 0.82 | (0.80, 0.84)* | | 0.90 | (0.88, 0.91)* | 1.02 | (1.00, 1.04)* |
| Triage Level (reference=5-Non-urgent) | |  |  |  |  |  |  |  |  | |  |  |  |  |
|  | 1- Resuscitation | 0.17 | (0.17, 0.18)* | 0.20 | (0.20, 0.21)* | 33.35 | (31.73, 35.37)* | 45.69 | (43.44, 48.09)* | |  |  |  |  |
|  | 2-Emergent | 0.40 | (0.40, 0.40)* | 0.40 | (0.40, 0.41)* | 11.83 | (11.25, 12.51)* | 20.05 | (19.04, 20.82)* | |  |  |  |  |
|  | 3-Urgent | 0.56 | (0.55, 0.56)* | 0.58 | (0.57, 0.58)* | 6.88 | (6.53, 7.25)* | 11.69 | (11.11, 12.15)* | |  |  |  |  |
|  | 4-Semi-urgent | 0.86 | (0.85, 0.86)* | 0.87 | (0.86, 0.87)* | 2.13 | (2.02, 2.26)* | 3.33 | (3.17, 3.48)* | |  |  |  |  |
|  | Missing | 1.01 | (0.98, 1.03) | 0.88 | (0.87, 0.89)* | 4.29 | (3.94, 4.77)* | 5.81 | (5.46, 6.15)* | |  |  |  |  |

Supplementary Table 9. Hazard ratios (HRs) and associated 95% confidence intervals (CIs) for multivariable models for the transitions to ED to community, ED to hospital, and hospital to community: variables related to diagnosis.

|  |  | ED to Community | | | | ED to Hospital | | | | Hospital to Community† | | | |
| --- | --- | --- | --- | --- | --- | --- | --- | --- | --- | --- | --- | --- | --- |
|  | | Control | | HSU | | Control | | HSU | | Control | | HSU | |
|  | | HR | (95% CI) | HR | (95% CI) | HR | (95% CI) | HR | (95% CI) | HR | (95% CI) | HR | (95% CI) |
| AIDS/HIV | | 0.31 | (0.23, 0.40)* | 0.41 | (0.34, 0.50)* | 1.24 | (0.85, 2.01) | 2.10 | (1.86, 2.43)* | 0.45 | (0.39, 0.53)* | 0.61 | (0.57, 0.65)* |
| Cancer | | 0.45 | (0.44, 0.46)* | 0.63 | (0.62, 0.64)* | 1.32 | (1.28, 1.36)* | 1.35 | (1.31, 1.38)* | 0.68 | (0.67, 0.69)* | 0.70 | (0.69, 0.71)* |
| Chest Pain | | 1.32 | (1.31, 1.32)* | 1.51 | (1.50, 1.52)* | 0.14 | (0.14, 0.15)* | 0.24 | (0.24, 0.25)* | 2.82 | (2.73, 2.91)* | 2.54 | (2.46, 2.61)* |
| Chronic pulmonary disease | | 1.36 | (1.35, 1.37)* | 1.40 | (1.39, 1.41)* | 0.98 | (0.96, 1.00)* | 1.25 | (1.23, 1.27)* | 1.14 | (1.12, 1.15)* | 1.24 | (1.23, 1.26)* |
| Congestive heart failure | | 0.39 | (0.38, 0.39)* | 0.57 | (0.56, 0.58)* | 1.19 | (1.16, 1.21)* | 1.39 | (1.37, 1.41)* | 0.88 | (0.87, 0.89)* | 1.01 | (1.00, 1.02)* |
| Dementia | | 0.56 | (0.54, 0.58)* | 0.72 | (0.69, 0.74)* | 0.69 | (0.66, 0.73)* | 0.69 | (0.65, 0.73)* | 0.55 | (0.54, 0.56)* | 0.66 | (0.65, 0.68)* |
| Diabetes | | 0.87 | (0.85, 0.88)* | 0.95 | (0.93, 0.96)* | 1.13 | (1.10, 1.17)* | 1.42 | (1.38, 1.45)* | 0.92 | (0.90, 0.94)* | 0.95 | (0.93, 0.97)* |
| Fever | | 0.82 | (0.81, 0.83)* | 0.79 | (0.78, 0.81)* | 0.97 | (0.94, 1.00)* | 1.40 | (1.36, 1.43)* | 1.42 | (1.33, 1.54)* | 1.51 | (1.45, 1.57)* |
| Gastrointestinal | | 0.91 | (0.91, 0.91)* | 1.00 | (1.00, 1.01) | 0.27 | (0.26, 0.28)* | 0.45 | (0.44, 0.45)* | 1.61 | (1.55, 1.69)* | 1.62 | (1.59, 1.65)* |
| General signs and symptoms | | 1.16 | (1.16, 1.16)* | 1.24 | (1.24, 1.25)* | 0.72 | (0.71, 0.73)* | 0.87 | (0.86, 0.88)* | 1.53 | (1.50, 1.56)* | 1.43 | (1.41, 1.44)* |
| Genitourinary/ obstetric | | 1.01 | (1.00, 1.01)* | 1.15 | (1.14, 1.15)* | 0.66 | (0.65, 0.66)* | 0.80 | (0.79, 0.81)* | 1.31 | (1.30, 1.33)* | 1.35 | (1.33, 1.36)* |
| Headache and other neurological | | 1.19 | (1.18, 1.19)* | 1.29 | (1.29, 1.30)* | 0.47 | (0.46, 0.47)* | 0.68 | (0.67, 0.68)* | 1.13 | (1.11, 1.15)* | 1.20 | (1.18, 1.21)* |
| Hemiplegia or paraplegia | | 0.47 | (0.42, 0.53)* | 0.49 | (0.45, 0.54)* | 0.94 | (0.82, 1.10) | 1.23 | (1.08, 1.38)* | 0.63 | (0.55, 0.74)* | 0.83 | (0.74, 0.93)* |
| Injury/trauma | | 1.55 | (1.55, 1.56)* | 1.58 | (1.58, 1.59)* | 0.61 | (0.60, 0.61)* | 0.65 | (0.64, 0.66)* | 1.06 | (1.05, 1.07)* | 1.05 | (1.04, 1.05)* |
| Liver | | 0.32 | (0.30, 0.33)* | 0.44 | (0.42, 0.45)* | 1.44 | (1.37, 1.51)* | 1.82 | (1.76, 1.88)* | 0.51 | (0.49, 0.53)* | 0.79 | (0.77, 0.81)* |
| Mental health | | 0.92 | (0.92, 0.93)* | 0.99 | (0.99, 1.00)* | 0.32 | (0.32, 0.33)* | 0.32 | (0.32, 0.33)* | 0.66 | (0.65, 0.67)* | 1.03 | (1.01, 1.04)* |
| Metastatic solid tumour | | 0.33 | (0.31, 0.35)* | 0.57 | (0.55, 0.59)* | 1.27 | (1.21, 1.33)* | 1.29 | (1.25, 1.34)* | 0.58 | (0.56, 0.59)* | 0.72 | (0.70, 0.73)* |
| Myocardial infarction | | 0.10 | (0.10, 0.11)* | 0.13 | (0.12, 0.14)* | 2.63 | (2.53, 2.75)* | 2.38 | (2.28, 2.51)* | 1.25 | (1.24, 1.26)* | 1.21 | (1.19, 1.22)* |
| Peptic ulcer disease | | 0.76 | (0.73, 0.78)* | 0.90 | (0.86, 0.93)* | 0.90 | (0.84, 0.97)* | 0.99 | (0.92, 1.07) | 1.18 | (1.15, 1.21)* | 1.09 | (1.05, 1.13)* |
| Peripheral vascular disease | | 0.55 | (0.53, 0.58)* | 0.74 | (0.71, 0.78)* | 1.05 | (1.00, 1.11)* | 1.16 | (1.11, 1.23)* | 0.78 | (0.75, 0.81)* | 0.85 | (0.82, 0.88)* |
| Renal disease | | 0.52 | (0.49, 0.56)* | 0.69 | (0.66, 0.72)* | 1.26 | (1.16, 1.38)* | 1.35 | (1.27, 1.44)* | 0.64 | (0.59, 0.70)* | 0.75 | (0.72, 0.79)* |
| Rheumatic disease | | 1.04 | (1.00, 1.09) | 1.28 | (1.24, 1.34)* | 0.98 | (0.89, 1.10) | 1.21 | (1.11, 1.33)* | 0.79 | (0.73, 0.85)* | 0.85 | (0.79, 0.90)* |
| Shortness of breath | | 1.07 | (1.06, 1.08)* | 1.17 | (1.15, 1.18)* | 0.60 | (0.58, 0.62)* | 0.80 | (0.78, 0.82)* | 1.62 | (1.50, 1.76)* | 1.81 | (1.73, 1.90)* |
| Skin problems | | 1.63 | (1.62, 1.64)* | 1.48 | (1.47, 1.49)* | 0.56 | (0.55, 0.58)* | 0.75 | (0.74, 0.77)* | 0.94 | (0.92, 0.97)* | 0.99 | (0.97, 1.00) |
| Stroke | | 0.62 | (0.62, 0.63)* | 0.68 | (0.67, 0.70)* | 1.27 | (1.25, 1.29)* | 1.35 | (1.32, 1.38)* | 0.86 | (0.85, 0.87)* | 1.00 | (0.99, 1.02) |
| Upper respiratory infections / otitis media | | 2.02 | (2.01, 2.03)* | 2.32 | (2.30, 2.34)* | 0.17 | (0.16, 0.18)* | 0.23 | (0.22, 0.25)* | 2.05 | (1.89, 2.22)* | 2.00 | (1.91, 2.09)* |

†=hospital diagnosis

Supplementary Figure 1. Ratio of HSU vs control hazard ratios and associated 95% confidence intervals for the ED to death transition. Reference categories are Ontario, female, combined medium-low to highest income quintile, large urban area, and family physician or other health care provider.


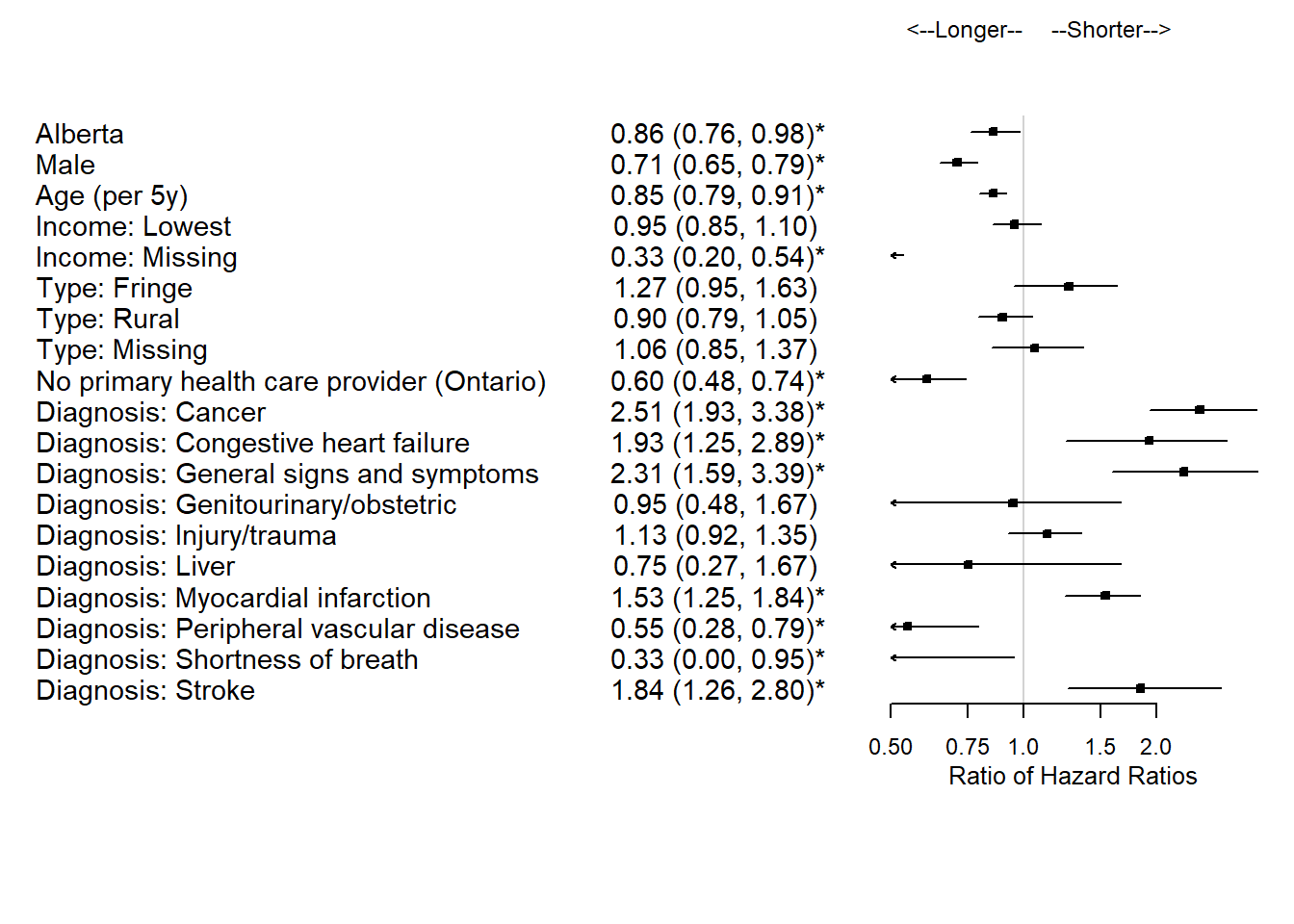


Supplementary Figure 2. Ratio of HSU vs control hazard ratios and associated 95% confidence intervals for the hospital to death transition. Reference categories are Ontario, female, combined medium-low to highest income quintile, large urban area, and family physician or other health care provider.


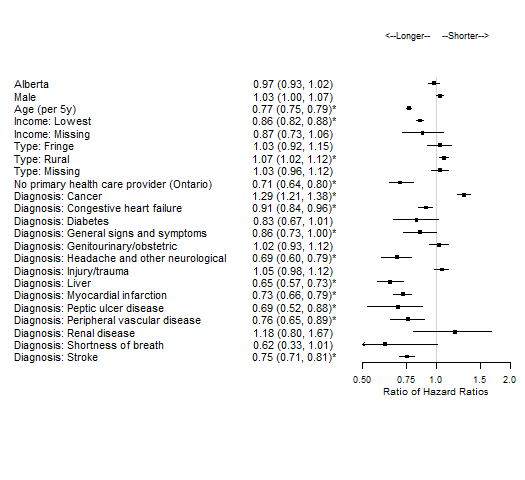


Supplementary Figure 3. Ratio of HSU vs control hazard ratios and associated 95% confidence intervals for the hospital to community transition (key diagnostic groups presented). Reference categories are Ontario, female, combined medium-low to highest income quintile, large urban area, family physician or other health care provider, and diagnoses other than those presented in Supplementary Table 9.


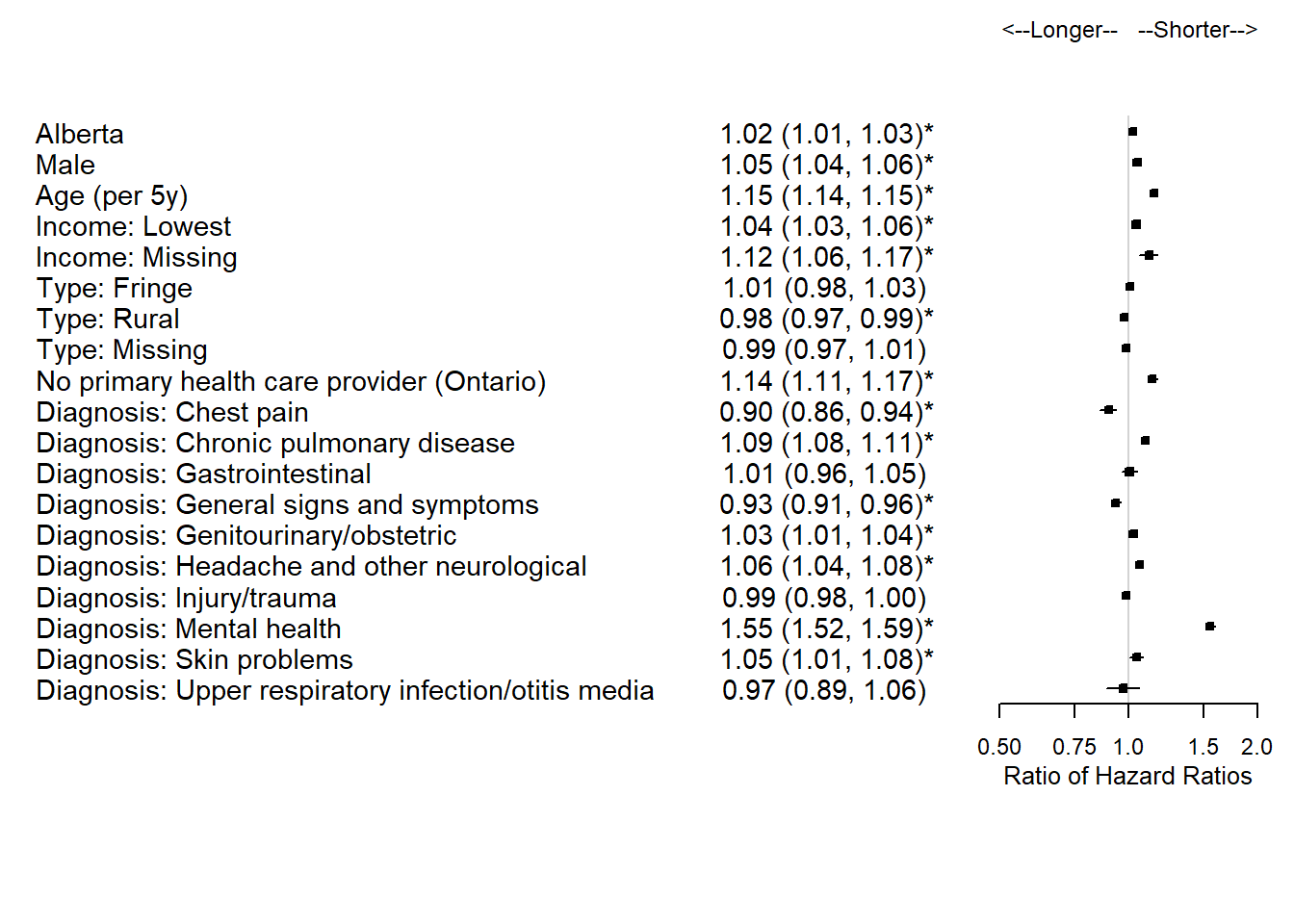


Supplementary Figure 4. Ratio of HSU vs control hazard ratios and associated 95% confidence intervals for the community to hospital transition. Reference categories are Ontario, female, combined medium-low to highest income quintile, large urban area, and family physician or other health care provider.

**
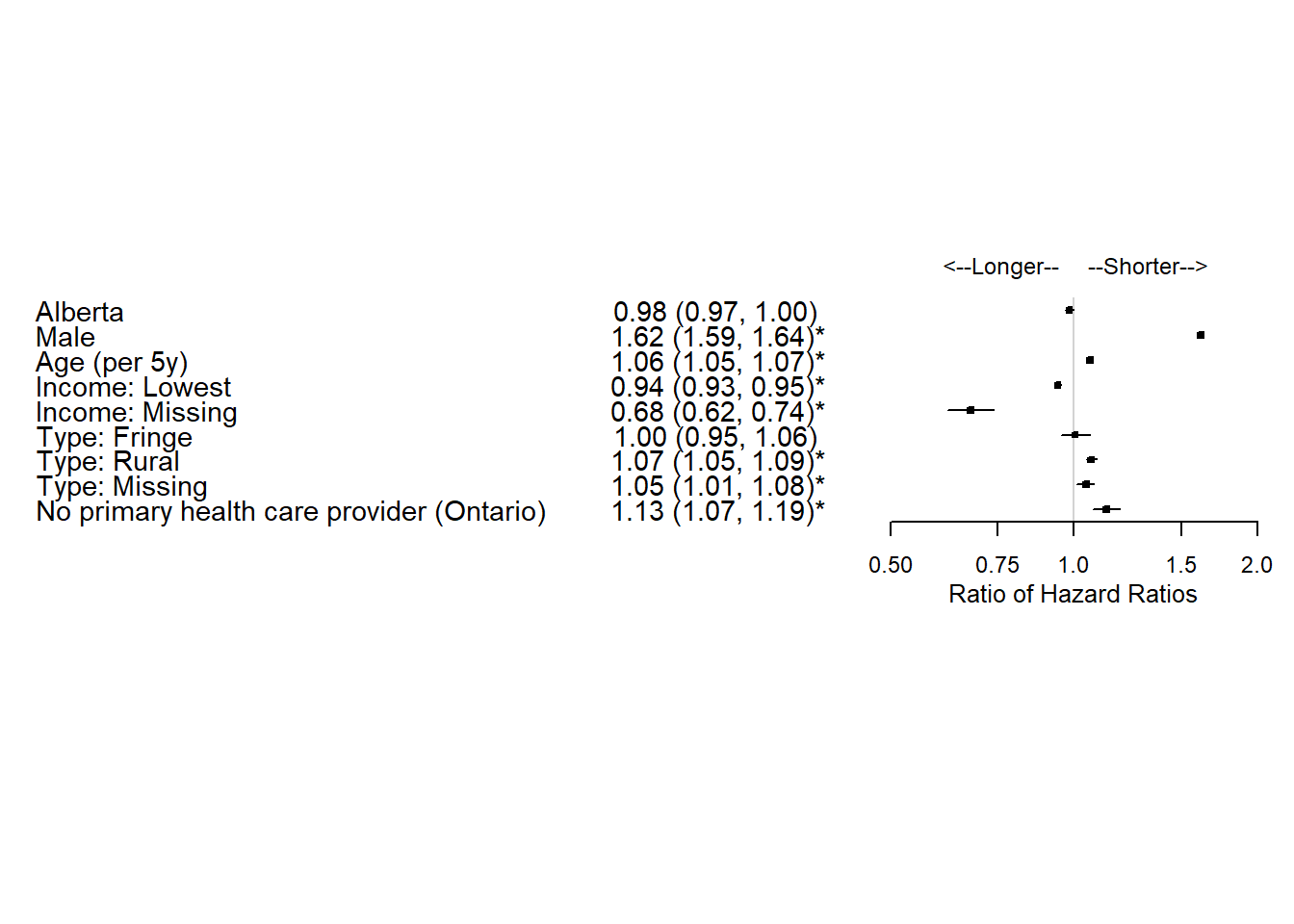
**

Supplementary Figure 5. Cummulative hazard function estimates for HSUs and controls (CON) for the different state transitions.

| 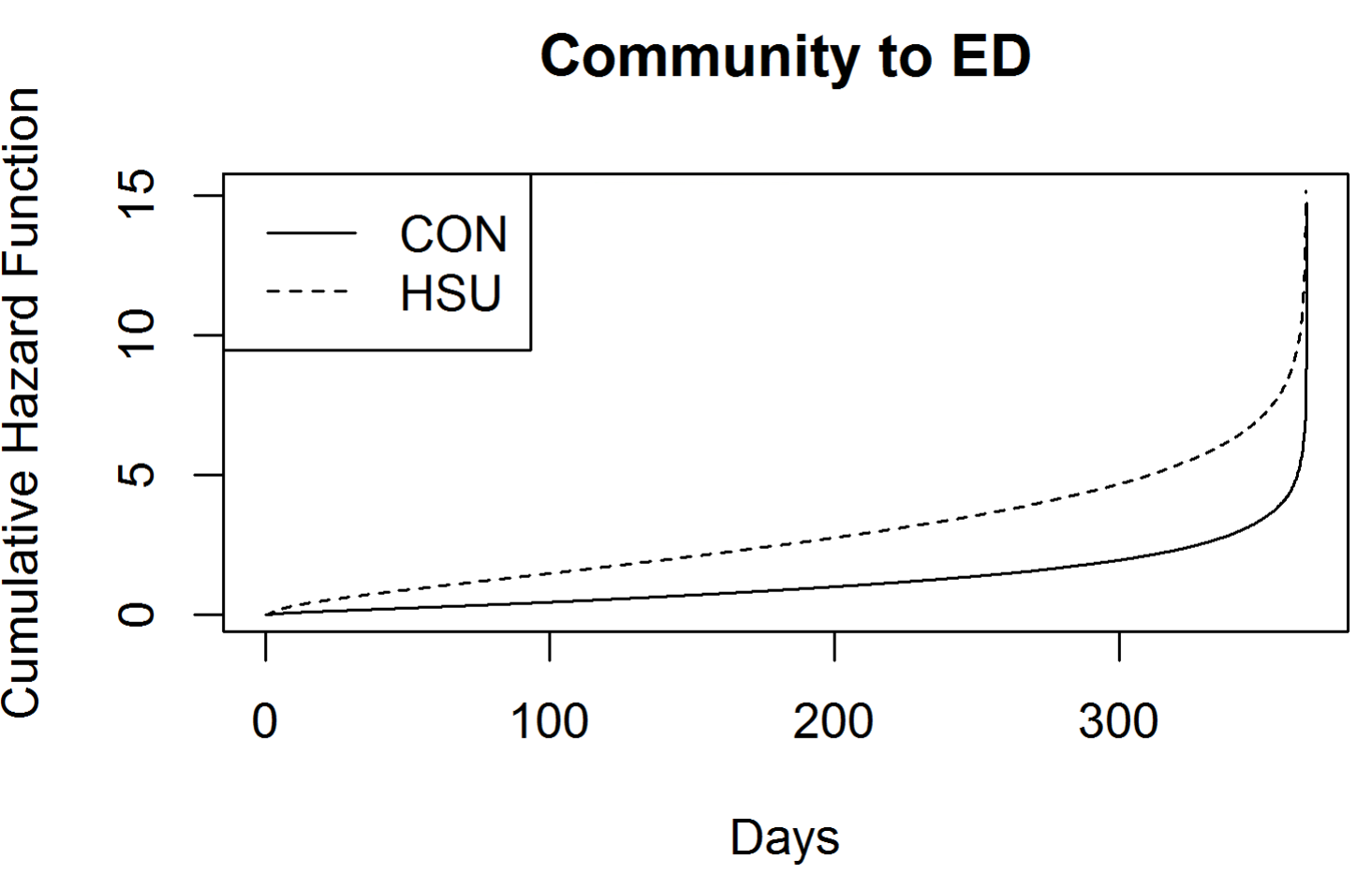 | 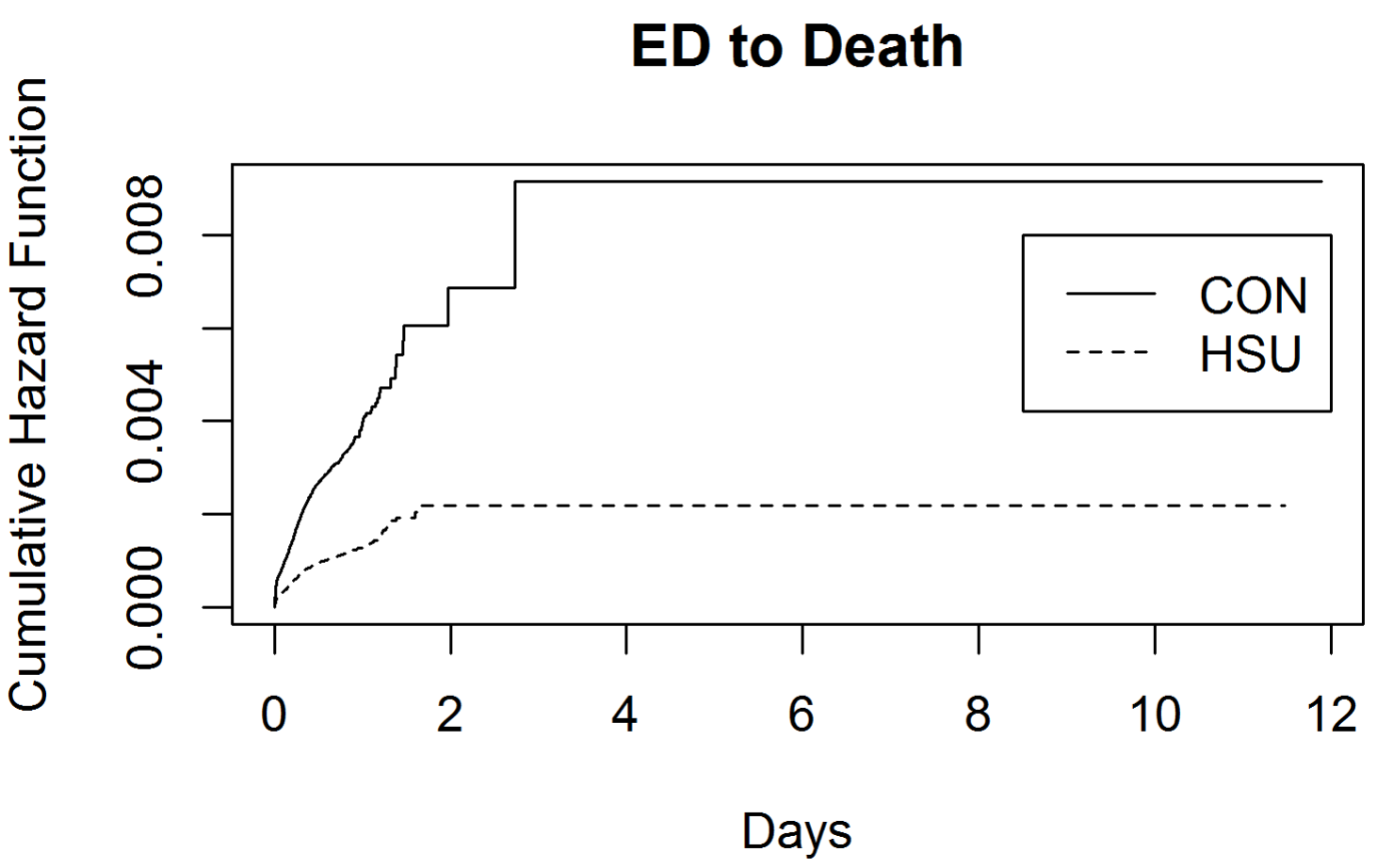 |
| --- | --- |
| 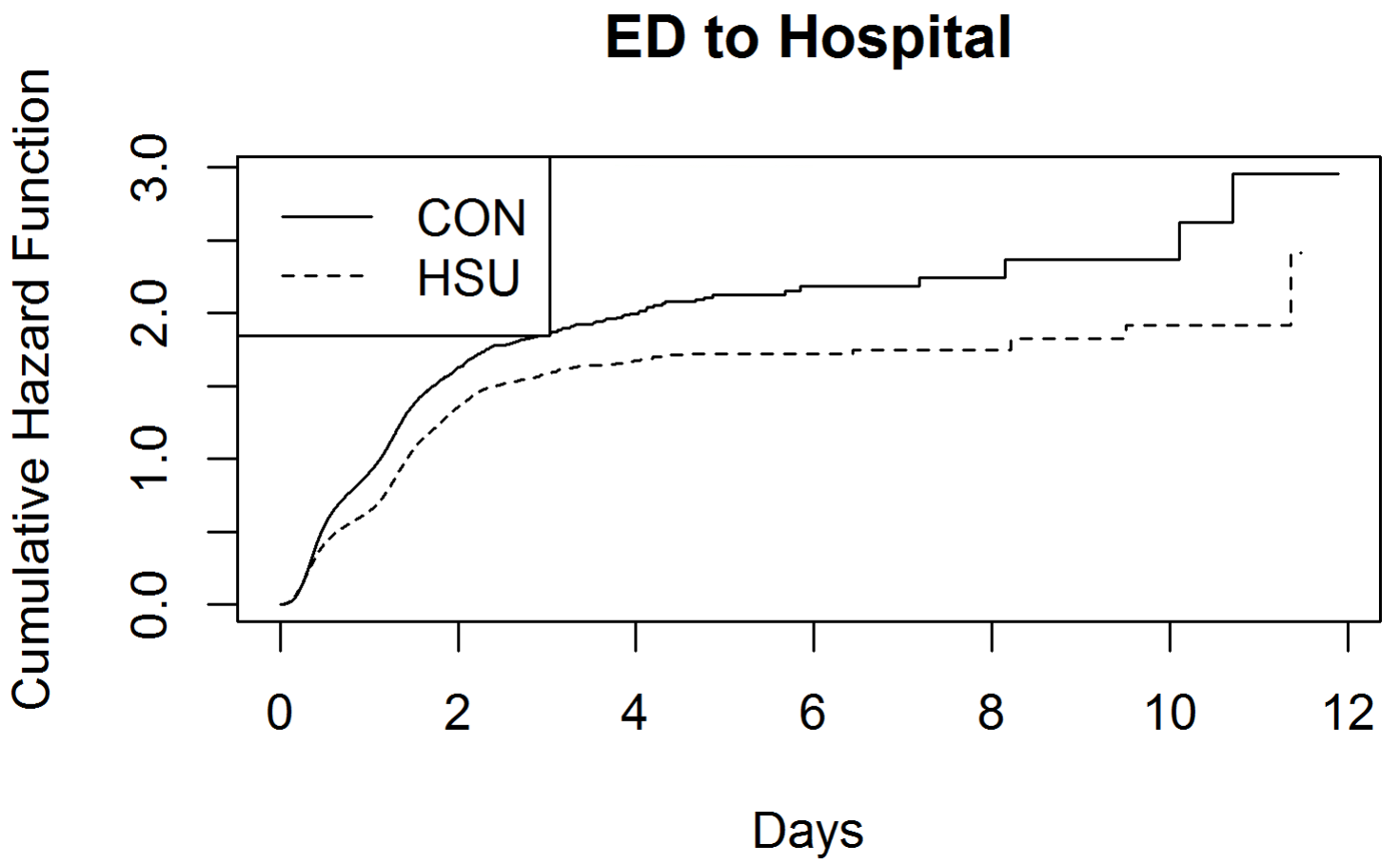 | 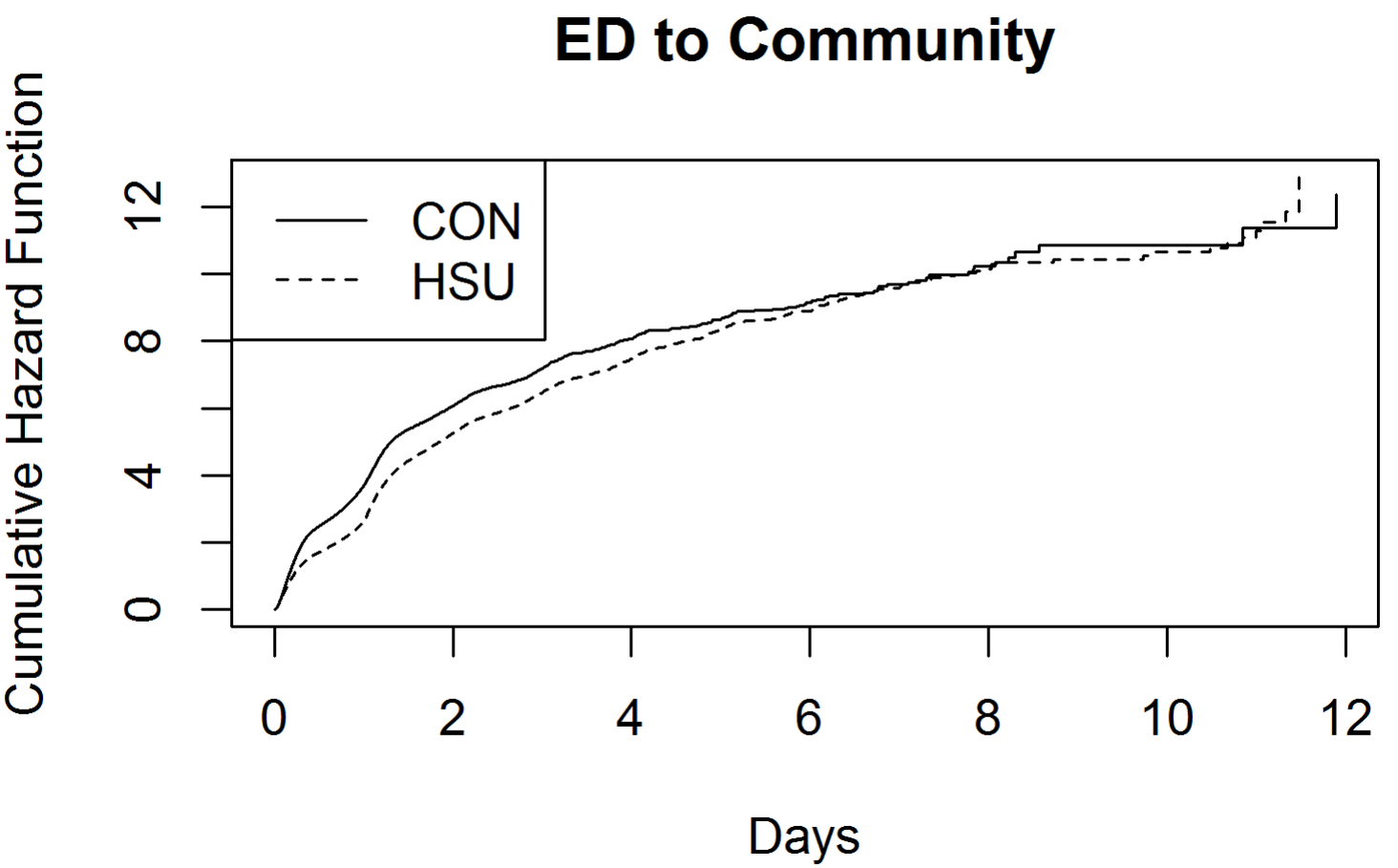 |
| 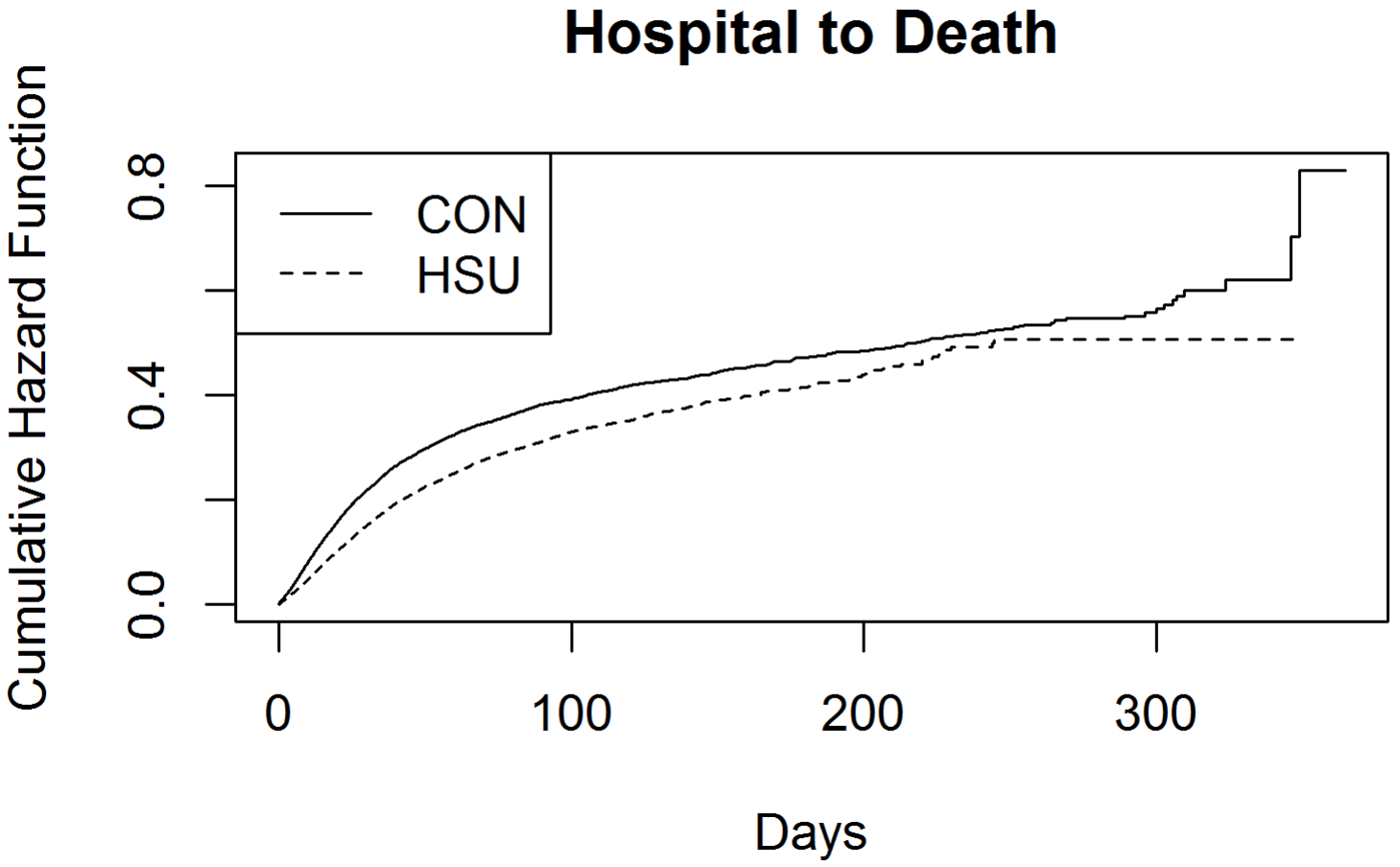 | 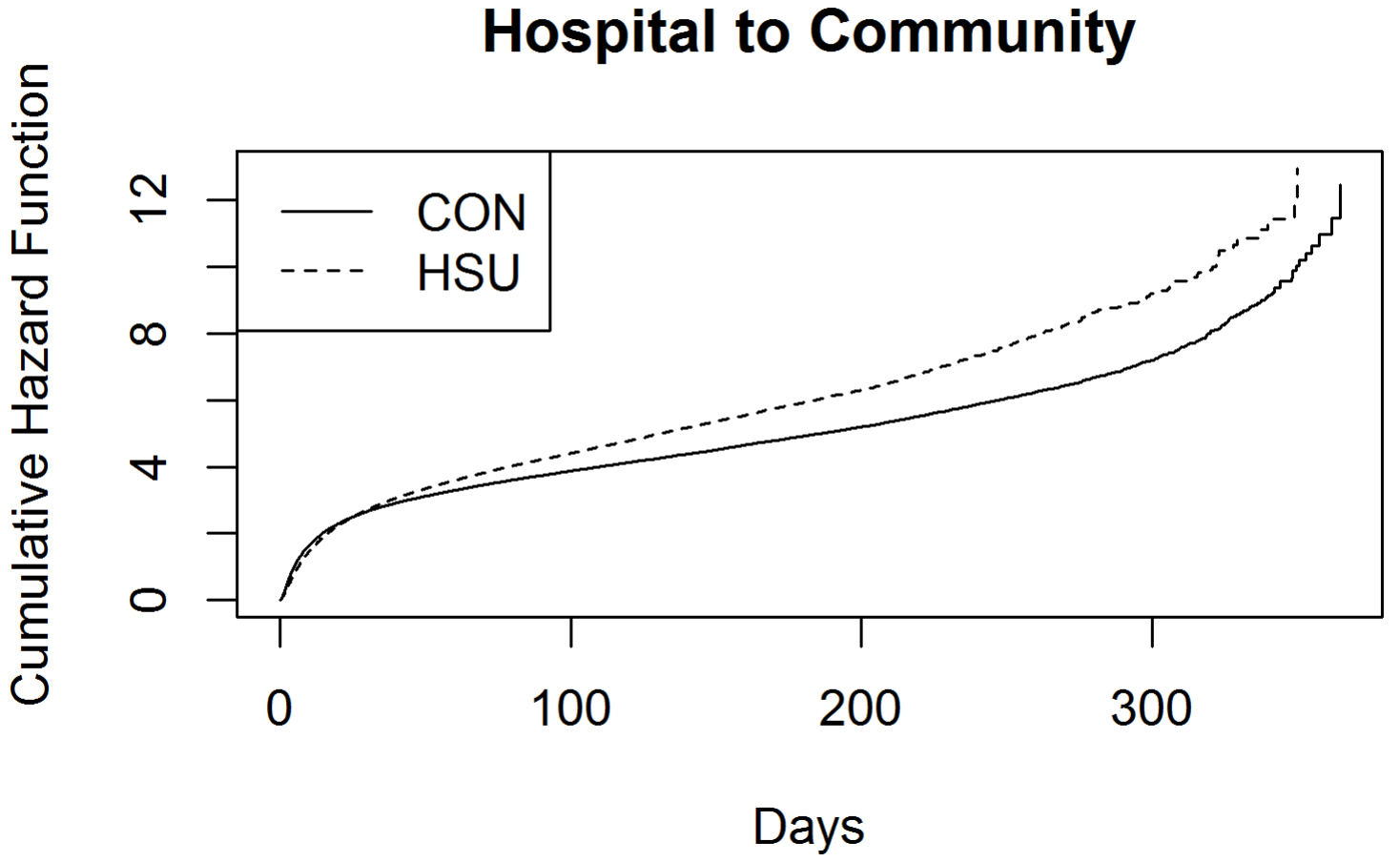 |
| 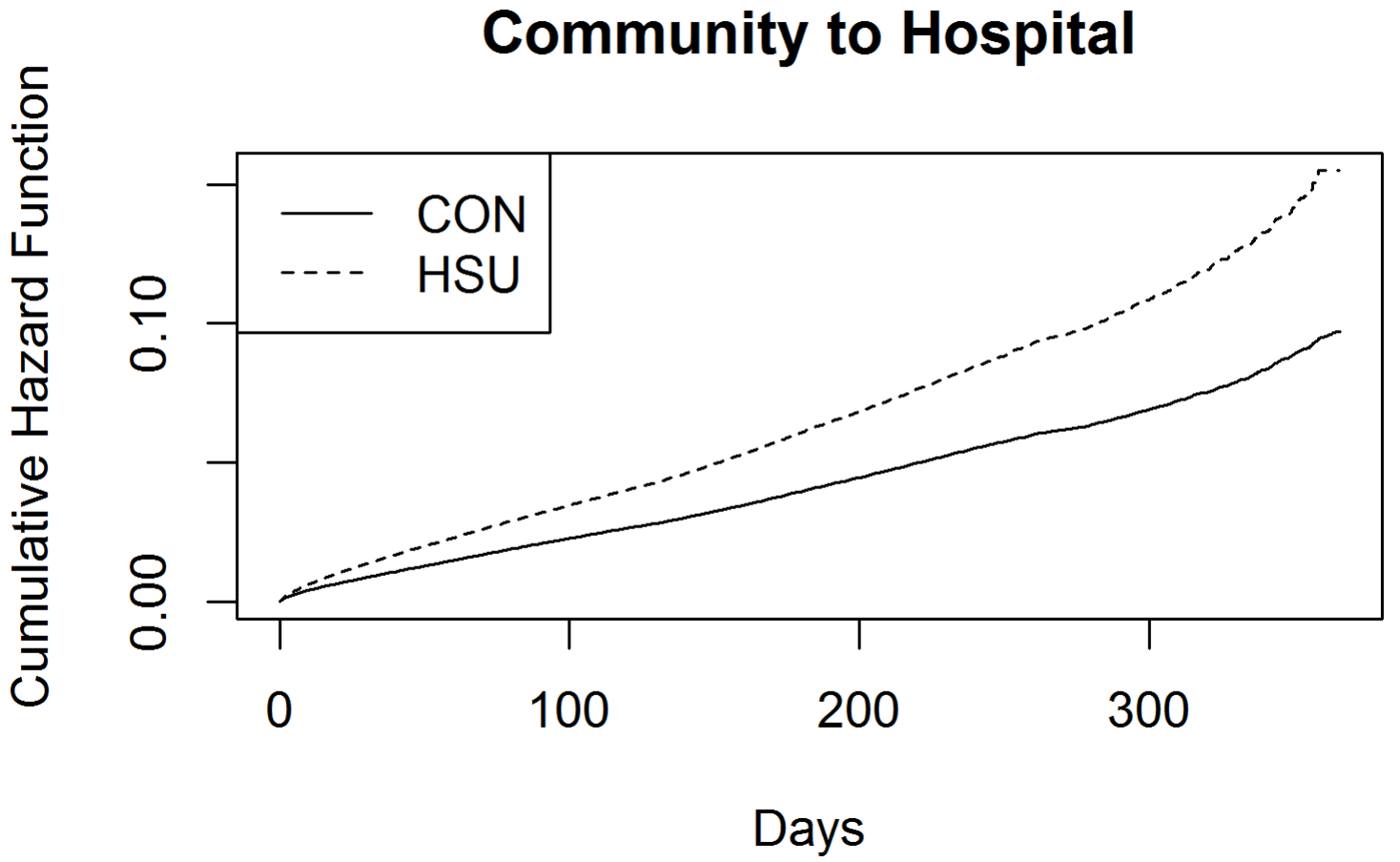 |  |
